# Development of a nutritional risk screening tool for preterm children in outpatient settings during a complementary feeding period: a pilot study

**DOI:** 10.1186/s12887-022-03774-5

**Published:** 2022-12-07

**Authors:** Xiaoying He, Zhuobin Jiang, Cuiling Wu, Lingyan Zeng, Meijiao Qi, Yalian Sun, Yanna Zhu

**Affiliations:** 1 Department of Child Healthcare, Affiliated Foshan Maternity and Child Healthcare Hospital, Southern Medical University (Foshan Maternity and Child Healthcare Hospital), Foshan, 528000 China; 2 Department of Maternal and Child Health, School of Public Health, Sun Yat-sen University, Guangzhou, 510080 China; 3 Information Centre, Affiliated Foshan Maternity and Child Healthcare Hospital, Southern Medical University (Foshan Maternity and Child Healthcare Hospital), Foshan, 528000 China

**Keywords:** Nutritional risk, Screening tool, Preterm, Complementary feeding

## Abstract

**Background:**

A complementary feeding (CF) period is necessary for nutritional and developmental reasons. Preterm children encounter more feeding problems than their term counterparts in the CF period. The goal of this study was to develop a nutritional risk screening tool specific to preterm children (the NRSP) in outpatient settings in the CF period, with the expectation of providing a standardised process to determine feeding problems and subsequently offering targeted nutritional advice.

**Methods:**

This study was a 2-phase study consisting of the development and evaluation phases. In the development phase, the items of the NRSP were initially developed based on references and the Delphi expert consultation method. Second, 329 preterm individuals with corrected ages from 5 to 36 months were enrolled. The participating preterm children were interviewed with the NRSP and anthropometric measurements, and underwent intellectual developmental tests and biochemistry detection (haemoglobin, red blood cell count, mean corpuscular volume, mean corpuscular haemoglobin, mean corpuscular haemoglobin concentration, serum iron, vitamin D). Third, preterm children’s anthropometric parameters were remeasured 1 month (for infants whose corrected age was 5–11 months) or 3 months (for children whose corrected age was 12–36 months) after the interview. Data in the development phase were analysed via univariate and binary logistic regression analysis sequentially to assign scores for items of the NRSP and to generate the models to predict underweight, stunting, and microcephaly of the NRSP. In the evaluation phase, another 605 preterm individuals were recruited to undergo the interview, anthropometric measurements, intellectual developmental tests, and biochemistry detection as in the development phase. Interrater reliability, test-retest reliability, area under the curve (AUC), accuracy, sensitivity, specificity, the positive/negative predictive value (P/NPV), the positive/negative likelihood ratio (LR+/−), and the correlation coefficient by Spearman’s correlation analysis (r_s_) were used to assess the reliability and validity of the NRSP. Finally, anthropometric parameters, biochemistry levels, and intellectual development quotients (DQs) from the development and evaluation phases between the high- and low-risk groups classified by the NRSP were compared using a t-test.

**Results:**

The κ coefficients of the interrater and test-retest reliability of the NRSP were all above 0.600, which meant that the reliability of the NRSP was moderate to substantial. The NRSP exhibited relatively higher efficiency in predicting underweight and stunting, with AUCs, accuracies, specificities, and NPVs near to or greater than 0.900, sensitivities above 0.600, PPVs above 0.400, LR + s near to or greater than 10, and r_s_s above 0.400. On the other hand, the NRSP manifested a weaker ability in predicting microcephaly, with most of the values of validity indicators lower than those of underweight and stunting prediction. Z scores of body weight, body length and head circumference, as well as DQs, were all higher in the low-risk groups than in the high-risk groups. There were no significant differences with respect to biochemistry levels between the high- and low-risk groups.

**Conclusion:**

The NRSP shows moderate to substantial reliability and validity in predicting underweight, stunting, and microcephaly. Health care staff should shed light on improving the feeding practices of preterm children with high nutritional risk classified by the NRSP to facilitate their physical growth and intellectual development. More research is expected to promote the NRSP models.

**Supplementary Information:**

The online version contains supplementary material available at 10.1186/s12887-022-03774-5.

## Introduction

Clearly, at the corrected age of 4–6 months, preterm infants enter a process when breast milk or formula alone is not sufficient for further nutritional requests such that complementary foods are needed [1, 2]. The appropriate timing of the introduction of complementary foods [3–5] and macronutrients and micronutrients provided by complementary foods [6–9] have positive effects on children’s (including preterm children’s) physical growth and cognitive development. In addition to its nutritional effect, food-related behaviours (such as satiety responsiveness, food fussiness), skills, and attitudes acquired during the complementary feeding (CF) period have long- and short-term health effects [10, 11]. Generally, the CF period is necessary for nutritional and developmental reasons.

However, preterm children encounter more feeding problems than their term counterparts in the CF period, such as improper use of nutritional fortifiers [12, 13], inappropriate timing of complementary food introduction [14, 15], failure in food diversity [16], delay in transition from liquid to solid foods [17], insufficiency in an energy dense diet [17] and feeding difficulty [18, 19]. For example, 21.64% of preterm infants introduced complementary foods earlier than the corrected age of 4 months, which was too early, and that rate was significantly higher than 1.71% of the term infants [17]. A total of 46.6% of preterm infants, compared to 35.0% of term infants, had feeding difficulty in the CF period [19].

Therefore, nutritional risk screening for preterm children in the CF period to provide further targeted nutritional advice is vital. However, current nutritional risk screening tools for children aim to rapidly identify hospitalised paediatric patients with high nutritional risk and initiate nutritional intervention to decrease the length of the hospital stay, morbidity and mortality, as well as hospital costs [20, 21]. The content of current nutritional risk screening tools consists of three or four questions that ask about whether there is potential nutritional risk of the current diseases, loss of nutritional intake, and poor weight gain [22]. Obviously, these questions do not cover enquiries into the feeding practices in the CF period.

The goal of our study was to develop a nutritional risk screening tool specific to preterm children (the NRSP) in outpatient settings during the CF period, expecting to provide a standardised process for child health care staff to determine the feeding problems of preterm individuals and subsequently to give targeted nutritional advice.

## Materials and methods

The study comprised two phases: the development and evaluation phases. The study protocol was approved by the Medical Ethics Committees of Affiliated Foshan Maternity and Child Healthcare Hospital, Southern Medical University (Foshan Maternity and Child Healthcare Hospital) (approval #FSFY-MEC-2020-028).

### Development phase

#### Development of NRSP

Professor Zhu from the Department of Maternal and Child Health at the School of Public Health at Sun Yat-sen University, and four medical staff qualified attending physicians in Foshan Maternity and Child Healthcare Hospital designed the item pool of the NRSP based on local nutritional recommendations [1], references on preterm children’s nutrition, current nutritional risk screening tools for paediatric patients, and work experience.

The structure of the NRSP was made up of four dimensions (Additional file 1: Appendix 1, Additional file 2: Appendix 2 and Additional file 3: Appendix 3).

The first dimension inquired about the preterm individual’s health status. The preterm individual was asked whether he/she was suffering from functional disorders, such as gastrointestinal, cardiopulmonary, neurological, haematological or metabolic disorders, as well as allergic or acute ailments [23–29].

The second dimension investigated feeding practices. Due to marked changes in the diet from the corrected age of 5 months to 3 years of age among preterm individuals [10], the second dimension of NRSP was divided into three parts specific to preterm children with a corrected age of 5–7 months, 8–11 months, and 1–3 years. The volume of milk intake, the kind of formula (e.g., nutritional fortifiers such as human milk fortifier and post-discharge formula, special formulae such as extensively hydrolysed formula and amino acid formula), the amount of cereal and animal food intake, and the frequency of different kinds of food (including red meat, white meat, egg and yolk, animal viscus, vegetables and fruits, and soybean products) were surveyed. The energy density of complementary food (whether the food served as a liquid, semisolid, or solid form) was also measured. Furthermore, the preterm individual was asked whether he/she had difficulty or choked when swallowing or if he/she was unwilling to eat, which was defined as perceived eating difficulty.

The third dimension addressed nutrient supplementation. Preterm children are susceptible to deficiencies in vitamin A and D, as well as iron or calcium [28, 30, 31]. Thus, we designed the items for this section to inquire about ‘vitamin A, vitamin D, and iron element supplement quantity per week’, ‘hours spent outdoors per week’, and ‘other nutrient supplements (e.g., calcium, zinc, etc.)’.

The last dimension involved anthropometric assessment. We inquired about whether there was foetal growth retardation (z-score of birth weight/length/head circumference < − 2) or recent poor physical growth (z-score of body weight/length/head circumference at present minus the z-score of body weight/length/head circumference from last time < − 0.2), since those two factors might indicate extrauterine growth retardation [18, 32].

Thereafter, eight experts were invited to assess the content validation of the item pool. One of eight experts majored in paediatrics, one in neonatology, one in nutriology, and the other five in child health care. The qualification of each expert was no less than associate doctor or associate professor, with at least 10 years of work experience. Experts used a 5-point Likert scale (1 = not important at all, 5 = very important) to establish the importance of each item on the NRSP. After two evaluations and revisions, all items of the NRSP received a score higher than four, meaning that all items were essential.

#### Generation of nutritional risk predicting models for NRSP

#### Participants

There were 18, 20, and 16 items of the NRSP for preterm children aged at corrected 5–7, 8–11, and 12–36 months, respectively. The sample size of each age group should be more than five times the number of items [33]. Hence, we needed to recruit no less than 100 preterm children for each age group. We recruited preterm children (gestational age < 37 weeks) with a corrected age of 5–36 months who underwent a physical examination in the Child Health Care Department of Foshan Maternity and Child Healthcare Hospital from March to July 2020. On the other hand, preterm children who were diagnosed with metabolic diseases and required a special diet were excluded. Preterm children with incomplete data were also excluded.

#### Investigation, anthropometry, and intellectual developmental and biochemical tests

Five well-trained medical staff members in the Child Health Care Department of Foshan Maternity and Child Healthcare Hospital conducted the investigation. After obtaining written informed consent from the caregivers, the medical staff interviewed the caregivers using the NRSP. After the interview, the participating preterm children were anthropometricly measured for body weight, body length, and head circumference and underwent intellectual developmental tests using the ‘Developmental Scale for Children Aged 0–6 years of China’. The intellectual developmental level was described as the development quotient (DQ, mental age/corrected age), including the gross motor index, fine motor index, adaptability index, verbal index, and social communication index; the full-scale DQ was the average of the five indices. For example, if the gross motor ability of a preterm infant with a corrected age of 8 months could reach the level of a term infant at 8 months old, his/her gross motor mental age was 8, and his/her gross motor DQ was 100 (8/8). A higher DQ indicated better potential for intellectual development. Subsequently, 5-ml venous blood samples were taken from the preterm children for haemoglobin, red blood cell (RBC) count, mean corpuscular volume (MCV), mean corpuscular haemoglobin (MCH), mean corpuscular haemoglobin concentration (MCHC), serum iron, and vitamin D detection. The blood samples were tested on the day of the interview. The haemoglobin, RBC count, MCV, MCH, and MCHC levels were detected by an automated haematology analyser XN-10, Sysmex Corporation. The levels of serum iron were tested by an automatic biochemical immunoassay analyser (Cobas 800, Roche Diagnostics Corporation). The levels of vitamin D were tested by a Mokosensor-A300 colloidal gold immunochromatographic analyser. Finally, preterm children’s anthropometric parameters were remeasured one month (for corrected 5–11-month age groups) or three months (for corrected 12–36-month age group) after the interview.

#### Nutritional risk definition

We defined underweight, stunting, or microcephaly as high nutritional risk. Underweight was defined as a z-score of body weight 1 month or 3 months after the interview < − 2. Stunting was defined as a z-score of body length 1 month or 3 months after the interview < − 2. Microcephaly was defined as a z-score of head circumference 1 month or 3 months after the interview < − 2. Z-scores of anthropometric parameters were calculated according to the World Health Organization (WHO) growth chart.

#### Models to predict nutritional risk

The responses to each item of the NRSP were cross-tabulated with underweight/stunting/microcephaly (or not) to identify risk factors for underweight/stunting/microcephaly. Items found to be risk factors for underweight/stunting/microcephaly were scored as 0.5, 1, 2, or 3; protective factors were scored as − 0.5, − 1 or − 2; otherwise, they were scored as 0. In addition, factors that did not present significance in the current study but were recognised in the literature as having impacts on underweight/stunting/microcephaly were scored similarly. We then combined significant factors recognised by either univariate analysis or references to generate models that would best predict underweight/stunting/microcephaly using binary logistic regression analysis and area under the curve (AUC) analysis. Simultaneously, the cut-off values were determined by Youden’s index.

### Evaluation phase

#### Participants, investigation, anthropometry, intellectual developmental and biochemical tests

From August 2020 to May 2021, we recruited preterm children for the reliability and validity assessment of the NRSP. The sample size, the inclusion and exclusion criteria for the participants, and the execution of the investigation, anthropometry, intellectual developmental tests, and biochemical detection were the same as those in the development phase.

#### Reliability

Interrater reliability and test-retest reliability were used to assess the reliability of the NRSP. The first 30 participants from each age group were interviewed on the same day by two health care workers independently to evaluate the interrater reliability. Another 30 participants from each age group were reinterviewed after a week to evaluate the test-retest reliability.

#### Validity

AUC, accuracy, sensitivity, specificity, positive predictive value (PPV), negative predictive value (NPV), positive likelihood ratio (LR+), negative likelihood ratio (LR-), and correlation coefficient by Spearman’s correlation analysis (r_s_) were used to assess the effectiveness of the NRSP in predicting underweight, stunting, and microcephaly.

The correlations between the scores of each dimension of the NRSP and underweight/stunting/microcephaly were analysed using binary logistic regression analysis to evaluate which dimension would more likely predict nutritional risk.

Anthropometric parameters 1 or 3 months after the first interview, biochemical marker levels, and DQs between high-risk groups (which were estimated to have a higher risk of underweight, stunting or microcephaly) and low-risk groups by the NRSP classification from both the development and evaluation phases were compared to estimate the validity of risk classification based on the NRSP.

### Statistical analysis

Statistical analysis was carried out using IBM SPSS, version 25.0. Normally distributed continuous data were described as the mean and standard deviation (SD) values and compared by one-way analysis of variance (ANOVA) or Student’s t-test, whereas nonnormally distributed continuous data were demonstrated as the median and interquartile range (IQR) values and compared by the Kruskal–Wallis test. At the same time, categorical data are presented as frequencies and percentages, and were compared using the chi-square test or Fisher’s exact test.

Binary logistic regression analysis was used to generate nutritional risk prediction models and analyse the correlations between the scores of each dimension of the NRSP and malnutrition. Youden’s index was applied to yield the cut-off point. The interrater reliability and test-retest reliability were assessed using Cohen’s κ statistics. The AUC, accuracy, sensitivity, specificity, PPV, NPV, LR+, LR-, and correlation coefficient by Spearman’s correlation analysis (r_s_) were calculated to assess the validity of the NRSP.

## Results

### Demographic data

In the development phase, we first recruited 120, 128, and 136 preterm children at the corrected ages of 5–7, 8–11, and 12–36 months, respectively. The final samples of corrected ages of 5–7, 8–11, and 12–36 months were 104, 110, and 115, and the response rates were 88.67, 85.94, and 84.56%, respectively. In the evaluation phase, we preliminarily enrolled 182, 206, and 330 participants at the corrected ages of 5–7, 8–11, and 12–36 months, respectively. The final samples were 154, 176, and 275 for the corrected age groups of 5–7, 8–11, and 12–36 months, respectively, and the response rates were 84.61, 85.44, and 83.33%, respectively. Reasons for exclusion were: (1) incomplete data: some visitors were not the participants’ caregivers and could not provide complete data on feeding practices; (2) refusal or inability to take part in the intellectual developmental or biochemical tests: some preterm individuals could not cooperate on the developmental tests because of falling asleep or sickness, and some caregivers considered their infants too vulnerable to have blood drawn; (3) the absence of anthropometric remeasurement: some participants had moved away from Foshan or were under the care of another medical centre. The details of the study sample and reasons for exclusion are outlined in Table 1.

**Table 1:** Study sample and reasons for exclusion

Phase	Development			Evaluation		
Corrected age (months)	5–7	8–11	12–36	5–7	8–11	12–36
First recruitment (n)	120	128	136	182	206	330
Incomplete data (n)	0	0	0	0	2	5
Refusal or inability to participate in the intellectual developmental or biochemical tests (n)	8	6	0	12	9	8
Absence of anthropometric remeasurement (n)	8	12	21	16	19	42
Final sample (n)	104	110	115	154	176	275
Response rate (%)	86.67	85.94	84.56	84.61	85.44	83.33

There were discrepancies in the gender distribution (corrected age group of 5–7 months), the z-scores of head circumference on the day of the interview (corrected age group of 5–7 months), the z-scores of body length on the day of the interview (corrected age groups of 8–11, and 12–36 months), and corrected age (corrected age group of 12–36 months) between the development and evaluation phases. However, when developing the NRSP, we used the z-scores of the anthropometric parameters as outcome indicators, which had adjusted sex and corrected age, meaning that the discrepancies of gender distribution and corrected age would not affect the results. Moreover, the preterm children were stratified by their z-scores of anthropometric parameters. It was suggested that the discrepancies in head circumference and body length would fully be taken into account and would not interfere with the final outcomes. Data are shown in Table 2.

**Table 2:** Demographic data of the recruited participants

Corrected age (months)	5 to 7			8 to 11			12 to 36		
Phase	Development	Evaluation	P	Development	Evaluation	P	Development	Evaluation	P
n	104	154		110	176		115	275	
Male n (%)^a^	72(69.23)	79(51.30)	0.008	60(54.54)	105(59.66)	0.986	64(55.65)	175(63.64)	0.140
Gestational age n (%)^a^			0.863			0.347			0.977
< 32 weeks	20(19.23)	26(16.88)		25(22.73)	45(25.57)		24(20.87)	56(20.36)	
32–34 weeks	20(19.23)	33(21.43)		17(15.45)	38(21.59)		24(20.87)	60(21.82)	
> 34 weeks	64(61.54)	95(61.69)		68(61.82)	93(52.84)		67(58.26)	159(57.82)	
BWTZ^b^	− 0.31(− 0.80,0.75)	−0.24(− 0.72,0.25)	0.415	−0.11(− 0.69,0.38)	−0.25(− 0.73,0.27)	0.815	− 0.40(− 0.84,0.22)	−0.09(− 0.77,0.46)	0.361
BLGZ^b^	−0.24(− 0.81,0.37)	−0.23(− 0.71,0.36)	0.309	−0.20(− 0.711,0.03)	−0.25(− 0.91,0.24)	0.661	−0.37(− 1.04,0.15)	−0.36(− 1.00,0.16)	0.629
BHCZ^b^	−0.60(− 1.00,0.13)	− 0.56(− 1.00,0.06)	0.908	− 0.35(− 1.29,0.13)	−0.56(− 1.00,0.06)	0.061	− 0.47(− 1.00,0.06)	−0.49(− 1.05,0.05)	0.724
Corrected age (months)^b^	5.40(5.07,6.13)	5.20(4.90,5.71)	0.072	9.57(8.05,10.47)	9.97(8.37,11.40)	0.151	17.20(14.03,24.37)	16.43(12.80,18.37)	0.016
WTZ1^b^	−0.06(− 0.77,0.70)	0.00(− 0.55,0.73)	0.159	−0.08(− 0.79,0.48)	−0.34(− 0.80,0.74)	0.375	−0.56(− 1.24,0.24)	−0.38(− 1.00,0.16)	0.527
LGZ1^b^	−0.01 (− 0.59,0.69)	0.16(− 0.59,0.71)	0.172	−0.20 (− 0.80,0.55)	−0.14(− 0.64,0.64)	0.039	−0.26(− 1.02,0.55)	−0.37(− 1.33,0.40)	0.048
HCZ1^b^	−0.28(− 0.74,0.56)	0.23(− 0.41,0.81)	0.050	−0.10(− 0.73,0.70)	−0.01(− 0.80,0.48)	0.500	−0.42(− 0.99,0.44)	0.05(− 1.03,0.59)	0.905
WTZ2^b^	− 0.28(− 1.16,0.40)	−0.18(− 0.89,0.60)	0.230	−0.41(− 0.80,0.29)	−0.24(− 0.84,0.66)	0.094	−0.47(− 1.19,0.19)	−0.49(− 1.12,0.25)	0.691
LGZ2^b^	−0.18(− 0.95,0.43)	0.07(− 0.60,0.76)	0.237	−0.11(− 1.05,0.73)	−0.22 (− 0.82,0.44)	0.352	−0.37(− 1.14,0.34)	−0.36(− 1.28,0.25)	0.908
HCZ2^b^	−0.20(− 0.95,0.55)	0.03(− 0.63,0.81)	0.219	0.05(− 0.91,0.70)	−0.20(− 0.94,0.50)	0.460	−0.32(− 0.96,0.53)	−0.28(− 1.14,0.41)	0.962
Current diseases n (%)^a^			0.173			0.765			0.240
Neurological disorders	2(1.92)	5(3.25)		1(0.91)	4(2.27)		3(2.61)	4(1.45)	
Cardiopulmonary disorders	0	2(1.30)		0	1(0.57)		2(1.74)	0	
Gastrointestinal disorders	0	2(1.30)		0	1(0.57)		0	0	
Haematological system diseases	2(1.92)	0		0	0		0	2(0.73)	
Acute diseases	3(2.88)	12(7.79)		9(8.18)	18(10.23)		7(6.09)	23(8.36)	
Allergic diseases	8(7.69)	10(6.49)		6(5.45)	11(6.25)		3(2.61)	6(2.18)	

^a^by chi-square test, ^b^presented as the median (interquartile range) by the Kruskal–Wallis test, *BWTZ/BLGZ/BHCZ* z-scores of birth weight/length/head circumference, *WTZ/LGZ/HCZ1* z-scores of body weight/length/head circumference on the day of the interview, *WTZ/LGZ/HCZ2* z-scores of body weight/length/head circumference 1 month (for infants whose corrected age was 5–11 months) or 3 months (for children with a corrected age of 12–36 months) after the interview

### Models to predict nutritional risks

According to the results of univariate analysis (Additional file 4: Appendix 4, Additional file 5: Appendix 5 and Additional file 6: Appendix 6), binary logistic regression analysis (Additional file 4: Appendix 4, Additional file 5: Appendix 5 and Additional file 6: Appendix 6), references and our group discussions, models to predict nutritional risk were developed as follows.

#### For preterm infants at the corrected age of 5–7 months

The model to predict underweight included factors of the z-scores for birth weight, the volume of milk intake per day, nutritional fortifier usage, the amount of cereal and animal food intake, food energy density, and recent poor weight gain. The model to predict stunting included factors of the z-scores for birth length, the volume of milk intake per day, nutritional fortifier usage, vitamin D and calcium supplementation, hours spent outdoors per week, and recent poor body length growth. The model to predict microcephaly included factors of the z-scores for birth weight and birth head circumference, the volume of milk intake per day, nutritional fortifier usage, the amount of animal food intake, vitamin D supplementation, hours spent outdoors per week, and recent poor head circumference growth.

#### For preterm infants at the corrected age of 8–11 months

The model to predict underweight involved factors of the z-scores for birth weight and birth length, the volume of milk intake per day, nutritional fortifier usage, the amount of cereal and animal food intake, food energy density, perceived eating difficulty, and recent poor weight gain. The model to predict stunting involved factors of the z-scores for birth weight and birth length, the volume of milk intake per day, nutritional fortifier usage, the amount of animal food intake, vitamin D, vitamin A and calcium supplementation, hours spent outdoors per week, and recent poor body length growth. The model to predict microcephaly involved factors of the z-scores for birth head circumference, the volume of milk intake per day, iron rich food (red meat, egg and yolk, and animal viscus) intake frequency, the amount of animal food intake, perceived eating difficulty, vitamin D supplementation, hours spent outdoors per week, and recent poor head circumference growth.

#### For preterm children at the corrected age of 12–36 months

The model to predict underweight covered factors of the z-scores for birth weight and birth length, the volume of milk intake per day, red meat intake frequency, the amount of cereal and animal food intake, food energy density, perceived eating difficulty, and recent poor weight gain. The model to predict stunting covered factors of the z-scores for birth length, the volume of milk intake per day, the amount of animal food intake, perceived eating difficulty, vitamin D and calcium supplementation, hours spent outdoors per week, and recent poor body length growth. The model to predict microcephaly covered factors of the z-scores for birth head circumference, egg and yolk intake frequency, the amount of cereal and animal food intake, perceived eating difficulty, vitamin D supplementation, hours spent outdoors per week, and recent poor head circumference growth.

### Reliability

The κ coefficients of the interrater reliability and the test-retest reliability of the NRSP were all above 0.600, which meant that the reliability of the NRSP was moderate to substantial. The data are outlined in Table 3.

**Table 3:** The Reliability and Validity of the Nutritional Risk Screening Tool for Preterm Children

Corrected Age (months)	5 to 7	8 to 11	12 to 36
Interrater reliability (κ)	0.667*	0.758*	0.889*
Test-retest reliability (κ)	0.760*	0.706*	0.609*
**Models to predict underweight**			
AUC(95%CI)	0.863 (0.634–1.000)*	0.953 (0.885–1.000)*	0.907 (0.805–1.000)*
Accuracy	0.954	0.954	0.909
Sensitivity	0.769	0.692	0.652
Specificity	0.972	0.976	0.933
Positive predictive value	0.714	0.692	0.469
Negative predictive value	0.979	0.975	0.967
Positive likelihood ratio	27.084	28.257	9.662
Negative likelihood ratio	0.237	0.315	0.373
r_s_	0.558*	0.490*	0.476*
**Models to predict stunting**			
AUC(95%CI)	0.894 (0.741–1.000)*	0.975 (0.940–1.000)*	0.924 (0.870–0.978)*
Accuracy	0.916	0.969	0.894
Sensitivity	0.636	0.636	0.828
Specificity	0.937	0.970	0.901
Positive predictive value	0.437	0.583	0.432
Negative predictive value	0.971	0.976	0.983
Positive likelihood ratio	10.102	21.003	8.328
Negative likelihood ratio	0.388	0.375	0.193
r_s_	0.405*	0.443*	0.511*
**Models to predict microcephaly**			
AUC(95%CI)	0.730 (0.492–0.967)*	0.729 (0.465–0.993)#	0.922 (0.860–0.983)*
Accuracy	0.714	0.710	0.902
Sensitivity	0.692	0.692	0.720
Specificity	0.716	0.712	0.920
Positive predictive value	0.184	0.161	0.474
Negative predictive value	0.962	0.967	0.970
Positive likelihood ratio	2.440	2.402	9.000
Negative likelihood ratio	0.430	0.432	0.304
r_s_	0.401*	0.355*	0.513*

*κ* Cohen’s κ statistics, *AUC* Area under the curve, *r*_*s*_ Correlation coefficient by Spearman’s correlation analysis, *: *P* < 0.05; # *P* < 0.10

### Validity

The NRSP exhibited relatively higher efficiency in predicting underweight and stunting, with AUCs, accuracies, specificities, and NPVs near to or greater than 0.900, sensitivities above 0.600, PPVs above 0.400, LR + s near to or greater than 10, and r_s_s above 0.400. On the other hand, the NRSP manifested a weaker ability in predicting microcephaly, with most of the values of validity indicators lower than those of underweight and stunting prediction. Nevertheless, the LR-s of all the predictive models were above 0.1, which suggests less satisfactory results. The data are displayed in Table 3.

We further explored the correlations between the scores of each dimension of the NRSP and malnutrition. We found that the scores of anthropometric assessment were positively correlated with malnutrition in all age groups, except in stunting and microcephaly for preterm infants with a corrected age of 5–7 months. Feeding practices gradually manifested their significantly positive effect as age increased. However, nutrient supplementation did not have a significant correlation with underweight, stunting, or microcephaly. Data are depicted in Table 4.

**Table 4:** Correlations of scores of the Nutritional Risk Screening Tool for Preterm Children with underweight/stunting/microcephaly

	Underweight		Stunting		Microcephaly	
	OR (95% CI)	P	OR (95% CI)	P	OR (95% CI)	P
**Corrected age of 5–7 months**						
Feeding practice score	0.787 (0.454–1.364)	0.393	0.342 (0.120–0.972)	0.044	0.773 (0.404–1.482)	0.438
Nutrient supplementation score	–	–	1.578 (0.620–4.019)	0.339	0.503 (0.129–1.965)	0.323
Anthropometric assessment score	2.516 (1.322–4.753)	0.004	1.376 (0.629–3.010)	0.424	1.467 (0.948–2.271)	0.085
**Corrected age of 8–11 months**						
Feeding practice score	0.996 (0.646–1.535)	0.986	0.739 (0.312–1.750)	0.492	1.165 (0.883–1.538)	0.280
Nutrient supplementation score	–	–	1.710 (0.776–3.767)	0.183	1.369 (0.675–2.776)	0.384
Anthropometric assessment score	2.451 (1.556–3.862)	< 0.001	5.879 (2.006–17.228)	0.001	1.795 (1.032–3.124)	0.038
**Corrected age of 12–36 months**						
Feeding practice score	1.474 (1.039–2.091)	0.030	1.571 (1.062–2.326)	0.024	2.158 (1.358–3.429)	0.001
Nutrient supplementation score	–	–	1.101 (0.629–1.928)	0.736	1.496 (0.703–3.183)	0.295
Anthropometric assessment score	2.186 (1.429–3.346)	< 0.001	2.376 (1.407–4.013)	0.001	1.664 (1.043–2.656)	0.033

Finally, we compared anthropometric, biochemical, and intellectual developmental indicators between the high and low nutritional risk groups. We found that the z-scores for body weight, body length, and head circumference 1 or 3 months after the first interview were all greater in the low-risk groups versus the high-risk groups. Notwithstanding, there were no significant differences with respect to levels of haemoglobin, RBC count, MCV, MCH, MCHC, serum iron, and vitamin D between the high- and low-risk groups. The full-scale DQs of the high-risk groups were all lower than those of the low-risk groups. Gross motor and social communication DQs were lower in the high-risk group for preterm children with a corrected age of 12–36 months. Fine motor and adaptability DQs were lower in high-risk groups for preterm infants at the corrected age of 5–7, and 8–11 months. Verbal DQ was lower in the high-risk group for preterm infants at the corrected age of 8–11 months. Data are outlined in Table 5.

**Table 5:** The anthropometric parameters, biochemical levels, and intellectual development quotients between the high- and low-nutritional-risk groups

Corrected age	5–7 months			8–11 months			12–36 months		
Group	High nutritional risk	Low nutritional risk	P	High nutritional risk	Low nutritional risk	P	High nutritional risk	Low nutritional risk	P
n	83	175		65	221		99	291	
Male n (%)^a^	57 (68.67)	94 (53.71)	0.031	32 (49.23)	133 (60.18)	0.084	60 (60.61%)	179 (61.51)	0.873
GA (weeks)^b^	35.14 (32.71, 36.28)	35.14 (32.29, 36.14)	0.899	34.57 (32.00, 35.57)	34.57 (31.43, 35.96)	0.068	34.57 (32.43,35.86)	34.43 (32.57, 35.71)	0.763
WTZ2^b^	−0.51 (−1.43, −0.12)	0.01 (−0.69, 0.90)	< 0.001	−1.08 (−2.12, −0.58)	−0.12 (−0.66, 0.67)	< 0.001	−1.57 (−2.11, −0.79)	−0.41 (−0.96, 0.32)	< 0.001
LGZ2^b^	−0.36 (−1.08, 0.29)	0.16 (−0.56, 0.87)	0.003	−1.11 (−1.82, −0.28)	0.06 (−0.58, 0.82)	< 0.001	−1.37 (− 1.98, −0.30)	−0.27 (−0.92, 0.39)	< 0.001
HCZ2^b^	−0.34 (− 1.28, 0.22)	0.10 (− 0.58, 0.83)	0.002	− 1.03 (− 1.89, − 0.15)	0.19 (− 0.52, 0.73)	< 0.001	− 1.33 (− 2.19, − 0.52)	− 0.09 (− 0.75, 0.63)	< 0.001
Haemoglobin (g/L)^c^	117.40 (11.13)	115.80 (12.56)	0.385	119.09 (14.77)	120.88 (10.10)	0.361	124.70 (8.39)	126.11 (9.40)	0.235
RBC count (10^12^/L)^c^	4.76 (0.44)	4.76 (0.45)	0.977	4.90 (0.52)	4.79 (0.46)	0.174	4.82 (0.43)	4.99 (2.88)	0.593
MCV (fL)^c^	78.17 (6.98)	76.74 (9.90)	0.300	76.49 (9.10)	79.32 (8.42)	0.058	80.70 (8.14)	81.31 (8.00)	0.558
MCH (pg)^c^	24.81 (2.42)	24.48 (3.00)	0.436	24.52 (3.68)	25.43 (2.87)	0.085	26.02(2.60)	26.44 (2.71)	0.231
MCHC (g/L)^c^	317.41 (15.23)	317.26 (15.05)	0.947	319.51 (22.09)	320.64 (16.32)	0.713	322.61 (13.00)	325.25 (14.54)	0.151
Serum iron (μmol/L)^c^	11.79 (2.34)	11.25 (2.27)	0.166	11.02 (3.30)	12.13 (2.70)	0.187	12.91 (3.06)	13.41 (3.64)	0.444
Serum vitamin D (nmol/L)^c^	79.17 (14.67)	77.74 (14.07)	0.557	82.54 (12.91)	83.32 (11.80)	0.840	74.64 (17.37)	77.65 (17.51)	0.400
Gross motor DQ^c^	94.09 (17.25)	95.37 (12.91)	0.537	91.87 (12.15)	93.50 (10.90)	0.363	92.01 (12.45)	95.78 (11.49)	0.012
Fine motor DQ^c^	90.48 (15.27)	95.70 (14.16)	0.013	86.57 (10.60)	90.70 (10.99)	0.019	87.94 (11.55)	87.98 (12.83)	0.985
Adaptability DQ^c^	89.20 (13.03)	94.13 (12.12)	0.006	90.11 (12.04)	94.26 (10.03)	0.014	91.80 (11.05)	92.65 (11.33)	0.550
Verbal DQ^c^	95.67 (10.53)	98.60 (11.47)	0.068	88.53 (10.74)	92.61 (10.17)	0.014	83.28 (12.66)	85.99 (13.25)	0.104
Social communication DQ^c^	93.22 (12.26)	96.33 (11.00)	0.059	91.60 (10.48)	94.14 (9.03)	0.089	79.96 (11.26)	84.41 (10/83)	0.001
Full scale DQ^c^	92.57 (10.47)	96.03 (9.00)	0.012	90.18 (7.68)	93.04 (7.42)	0.017	86.99 (7.51)	89.39 (8.07)	0.017

^a^by chi-square test, ^b^presented as the median (interquartile range) by the Kruskal–Wallis test, ^c^presented as the mean (standard deviation) by Student’s t-test, *WTZ/LGZ/HCZ2* z-scores for body weight/length/head circumference 1 month (for infants with a corrected age of 5–11 months) or 3 months (for children with a corrected age of 12–36 months) after the interview, *RBC* Red blood cell, *MCV* Mean corpuscular volume, *MCH* Mean corpuscular haemoglobin, *MCHC* Mean corpuscular haemoglobin concentration, *DQ* Intellectual development quotient

## Discussion

The NRSP was designed for routine clinical use for health care staff when following up with preterm children. The data from this study indicate that the NRSP has acceptable reliability and validity.

### The NRSP has moderate to substantial reliability

We used interrater reliability and test-retest reliability to assess the ability of the NRSP in yielding the same nutrition outcome on the same individual. The interrater reliability and the test-retest reliability of the NRSP were all above 0.600, which implies that the reliability of the NRSP is moderate to substantial [34]. It is higher than the reliability of the Paediatric Yorkhill Malnutrition Score (PYMS, κ = 0.53) [35], the Screening Tool for Risk on Nutritional Status and Growth (STRONGkids, κ = 0.483), and the Paediatric Nutrition Screening Tool (PNST, κ = 0.601) [20], but lower than that of the Screening Tool for the Assessment of Malnutrition in Paediatrics (STAMP, κ = 0.882) [21].

### The NRSP has moderate to high validity

The AUCs of the NRSP in predicting underweight, stunting, and microcephaly were all above 0.700, which suggests that the effectiveness of the NRSP in malnutrition prediction is relatively high [36]. The AUCs of NRSP were greater than those of the PYMS and the STAMP in predicting wasting (0.717 and 0.657, respectively) and stunting (0.628 and 0.643, respectively) [37]. Sensitivities were all above 0.600, and specificities were all above 0.700 for NRSP, which indicates a moderate to high extent, and they were similar to the sensitivities of the PYMS and STAMP for predicting wasting (0.878 and 0.776, respectively) and stunting (0.724 and 0.759, respectively) [37]. Additionally, the PNST had an approximate sensitivity of 0.88 and a specificity of 0.78, while STRONGkids had a higher sensitivity of 0.94 but a lower specificity of 0.44 [20]. The accuracies of the NRSP in predicting underweight and stunting were near to or above 0.900, which were higher than those of the Subjective Global Nutritional Assessment (SGNA, 67.07%) and the STAMP (45.12%) [38]. The PPVs of the NRSP in predicting underweight and stunting were similar to the SGNA (64.86%) and STAMP (47.06%), while the NPVs of the NRSP were clearly higher than the SGNA (68.89%) and STAMP (47.06%). At the same time, LR + s were higher than the SGNA (2.14) and STAMP (0.93). On the other hand, LR-s were similar to the SGNA (0.52), but lower than the STAMP (1.33) [38]. For a disease with a 10% prevalence, the ideal sensitivity is 90%, specificity is 80%, PPV is 33%, NPV is 98%, LR+ is more than 10, and LR- is less than 0.1 for a diagnostic test [39]. In our study, the total prevalence of malnutrition was 11.43%. Hence, the NRSP has ideal specificity, PPV, NPV, and LR+ in predicting underweight and stunting; however, the NRSP’s potential to predict microcephaly is weaker; moreover, sensitivity and LR- are less favourable for the NRSP. The data revealed that the NRSP classifications were moderately correlated with underweight, stunting and microcephaly, with correlation coefficients varying from 0.355 to 0.558 [40]. This was slightly stronger for the NRSP associated with underweight than stunting or microcephaly, which was the same as reports of the STRONGkids (*r* = − 0.16 for weight for age, W/A; *r* = 0.03 for height for age, H/A) [41], SGNA (*r* = 0.440 for W/A, *r* = 0.278 for H/A) [42], and PNST (*r* = 0.66 for W/A, *r* = 0.19 for H/A) [43]. Further, the NRSP had a stronger correlation with the anthropometry than the STRONGkids, SGNA and PNST. The somewhat weaker validity and correlation of the NRSP with stunting or microcephaly versus underweight might be because body length or head circumference growth are significantly affected by genetic factors, the social and economic environment, cerebral development, and skull thickness compared to mere nutrition factors [44–46].

The z-scores for anthropometric parameters and intellectual DQs were significantly higher in the low-risk groups than in the high-risk groups, which indicates that the classification by the NRSP is valid and reasonable. Health care staff should shed light on improving the feeding practices of preterm children with high nutritional risk to facilitate their physical growth and intellectual development. There were no discrepancies with respect to haemoglobin, RBC count, MCV, MCH, MCHC, serum iron, and vitamin D levels between the high- and low-risk groups, which might be because this study was a single-centre investigation, and the participants basically followed the same advice on nutrient supplementation.

### Foetal growth status and feeding practices were critical factors for predicting malnutrition

We found that the anthropometric assessment score was positively correlated with malnutrition. The main component of the anthropometric assessment dimension was foetal growth status; therefore, we posited that foetal growth status was of paramount effect on extrauterine growth. A high score for anthropometric assessment indicates worse foetal growth status, which further signals less nutrient storage and a greater probability of disease occurrence, leading to extrauterine growth retardation.

The feeding practice score had a positive correlation with malnutrition only in the corrected 12–36-month age group, and even showed a negative association with malnutrition in the corrected 5–7-month age group. Reasons for the contrary relationship in the early stage might be that in the early stage of preterm birth, because of their low birth weight or length, they are probably regarded as having malnutrition, and the lower their birth weight is, the more likely they are to use nutritional fortifiers. When using nutritional fortifiers, preterm infants obtain lower scores in feeding practices, resulting in a false negative association between the score of feeding practices and malnutrition. As age increases, the effect of foetal growth status might be attenuated, and the positive effect of feeding practices gradually appears.

In the NRSP, some items (such as current diseases and nutrient supplementation, which are recognised in the literature as important factors of nutritional risk prediction) had no significant association with malnutrition in our study, which is contrary to our knowledge. We suspect that this was due to the relatively small sample size from a single centre. We decided to retain these items in the NRSP and anticipate further investigation.

There are limitations in this study. A major limitation was the relatively small sample size. This may have led to the second limitation, which was the moderate reliability and validity of the NRSP. Hence, a large-scale multicentre study should be conducted to broadly promote the NRSP models.

## Conclusion

The present study shows that the NRSP has moderate to substantial reliability and validity in predicting underweight, stunting, and microcephaly. Health care staff should shed light on improving the feeding practices of preterm children with high nutritional risk classified by the NRSP to facilitate their physical growth and intellectual development. However, more research is needed to promote the NRSP models.

## Supplementary Information

**Additional file 1: Appendix 1**. Nutritional Risk Screening Tool for Preterm Infants at the Corrected Age of 5–7 Months.

**Additional file 2: Appendix 2**. Nutritional Risk Screening Tool for Preterm Infants at the Corrected Age of 8–11 Months.

**Additional file 3: Appendix 3**. Nutritional Risk Screening Tool for Preterm Children at the Corrected Age of 12–36 Months.

**Additional file 4: Appendix 4–1**. Univariate analysis of responses to the screening tool and the z-scores classification of body weight, length and head circumference for preterm infants at the corrected age of 5–7 months [n (%)]. **Appendix 4–2**. Binary logistic regression analysis of responses to the screening tool and the z-scores classification of body weight, length and head circumference for preterm infants at the corrected age of 5–7 months (*P* > 0.900 were not shown).

**Additional file 5: Appendix 5–1**. Univariate analysis of responses to the screening tool and the z-scores classification of body weight, length and head circumference for preterm infants at the corrected age of 8–11 months [n (%)]. **Appendix 5–2**. Binary logistic regression analysis of responses to the screening tool and the z-scores classification of body weight, length and head circumference for preterm infants at the corrected age of 8–11 months (*P* > 0.900 were not shown).

**Additional file 6: Appendix 6–1**. Univariate analysis of responses to the screening tool and the z-scores classification of body weight, length and head circumference for preterm children at the corrected age of 12–36 months [n (%)]. **Appendix 6–2**. Binary logistic regression analysis of responses to the screening tool and the z-scores classification of body weight, length and head circumference for preterm children at the corrected age of 12–36 months (*P* > 0.900 were not shown).

## Data Availability

All data generated or analysed during this study are included in this article. Further inquiries can be directed to the corresponding author.

## Abbreviations

CF: Complementary feeding
NRSP: Nutritional risk screening tool for preterm children
AUC: Area under the curve
PPV: Positive predictive value
NPV: Negative predictive value
LR+: Positive likelihood ratio
LR-: Negative likelihood ratio
r_s_: Correlation coefficient by Spearman’s correlation analysis
DQ: Intellectual development quotient
RBC: Red blood cell
MCV: Mean corpuscular volume
MCH: Mean corpuscular hemoglobin
MCHC: Mean corpuscular hemoglobin concentration
WHO: World Health Organization
SD: Standard deviation
ANOVA: One-way analysis of variance
IQR: Interquartile range
PYMS: Paediatric Yorkhill Malnutrition Score
STRONGkids: Screening Tool for Risk on Nutritional Status and Growth
PNST: Pediatric Nutrition Screening Tool
STAMP: Screening Tool for the Assessment of Malnutrition in Paediatrics
SGNA: Subjective Global Nutritional Assessment
W/A: Weight for age
H/A: Height for age

## References

[1] Editorial board of Chinese Journal of Pediatrics, Pediatric health care panel of Chinese Medical Association, neonatology panel of Chinese Medical Association (2016). Feeding suggestions for premature and low birth weight infants after discharge. Chin J Pediatr.

[2] WHO (World Health Organization) (2002). Complementary Feeding. Report of the Global Consultation. Geneva, 10–13 December 2001. Summary of Guiding Principles.

[3] Marriott LD, Foote KD, Bishop JA, Kimber AC, Morgan JB (2003). Weaning preterm infants: a randomised controlled trial. Arch Dis Child Fetal Neonatal Ed.

[4] Spiegler J, Eisemann N, Ehlers S, Orlikowsky T, Kannt O, Herting E, Göpel W, GNN. (2015). Length and weight of very low birth weight infants in Germany at 2 years of age: does it matter at what age they start complementary food?. Eur J Clin Nutr.

[5] Sun C, Foskey RJ, Allen KJ, Dharmage SC, Koplin JJ, Ponsonby AL, Lowe AJ, Matheson MC, Tang ML, Gurrin L, Wake M, Sabin M (2016). The impact of timing of introduction of solids on infant body mass index. J Pediatr.

[6] Morgan J, Taylor A, Fewtrell M (2004). Meat consumption is positively associated with psychomotor outcome in children up to 24 months of age. J Pediatr Gastroenterol Nutr.

[7] Engelmann MD, Sandström B, Michaelsen KF (1998). Meat intake and iron status in late infancy: an intervention study. J Pediatr Gastroenterol Nutr.

[8] Neumann CG, Bwibo NO, Murphy SP, Sigman M, Whaley S, Allen LH, Guthrie D, Weiss RE, Demment MW (2003). Animal source foods improve dietary quality, micronutrient status, growth and cognitive function in Kenyan school children: background, study design and baseline findings. J Nutr.

[9] Whaley SE, Sigman M, Neumann C, Bwibo N, Guthrie D, Weiss RE, Alber S, Murphy SP (2003). The impact of dietary intervention on the cognitive development of Kenyan school children. J Nutr.

[10] Fewtrell M, Bronsky J, Campoy C, Domellöf M, Embleton N, Fidler-Mis N, Hojsak I, Hulst JM, Indrio F, Lapillonne A, Molgaard C (2017). Complementary feeding: a position paper by the European Society for Paediatric Gastroenterology, Hepatology, and nutrition (ESPGHAN) committee on nutrition. J Pediatr Gastroenterol Nutr.

[11] Boswell N (2021). Complementary feeding methods-a review of the benefits and risks. Int J Environ Res Public Health.

[12] Lu Y, Mao M, Yang F (2019). Issues regarding breastfeeding in preterm infants. Chin J Pediatr.

[13] Xiang Y, Tang QY (2019). Application of breast milk fortifier in breast feeding of very low birth weight infants. J Clin Pediatr.

[14] Braid S, Harvey EM, Bernstein J, Matoba N (2015). Early introduction of complementary foods in preterm infants. J Pediatr Gastroenterol Nutr.

[15] Vissers KM, Feskens EJM, van Goudoever JB, Janse AJ (2018). The timing of initiating complementary feeding in preterm infants and its effect on overweight: a systematic review. Ann Nutr Metab..

[16] Hambidge KM, Sheng X, Mazariegos M, Jiang T, Garces A, Li D, Westcott J, Tshefu A, Sami N, Pasha O, Chomba E, Lokangaka A, Goco N, Manasyan A, Wright LL, Koso-Thomas M, Bose C, Goldenberg RL, Carlo WA, McClure EM, Krebs NF (2011). Evaluation of meat as a first complementary food for breastfed infants: impact on iron intake. Nutr Rev.

[17] Sun YL, He XY, Wu CL, Liu K, Ma J (2019). Feeding status of preterm infants during the food conversion period. J Nur Sci.

[18] Medoff-Cooper B, Rankin K, Li Z, Liu L, White-Traut R (2015). Multisensory intervention for preterm infants improves sucking organization. Adv Neonatal Care.

[19] Sun YL, He XY, Zeng JY, Wu CL, Liu K, Zeng LY (2020). Preliminary study on responsive feeding of preterm infants during food conversion period. Chin J Child Health Care.

[20] Carter LE, Shoyele G, Southon S, Farmer A, Persad R, Mazurak VC, BrunetWood MK (2020). Screening for pediatric malnutrition at hospital admission: which screening tool is best?. Nutr Clin Pract.

[21] McCarthy H, Dixon M, Crabtree I, Eaton-Evans MJ, McNulty H (2012). The development and evaluation of the screening tool for the assessment of malnutrition in Paediatrics (STAMP©) for use by healthcare staff. J Hum Nutr Diet.

[22] Hulst JM, Zwart H, Hop WC, Joosten KF (2010). Dutch national survey to test the STRONGkids nutritional risk screening tool in hospitalized children. Clin Nutr.

[23] Teresa C, Antonella D, de Ville DG (2019). New nutritional and Therapeutical strategies of NEC. Curr Pediatr Rev.

[24] Lenfestey MW, Neu J (2018). Gastrointestinal development: implications for Management of Preterm and Term Infants. Gastroenterol Clin N Am.

[25] Voynow JA (2017). "new" bronchopulmonary dysplasia and chronic lung disease. Paediatr Respir Rev.

[26] Luu TM, Rehman Mian MO, Nuyt AM (2017). Long-term impact of preterm birth: neurodevelopmental and physical health outcomes. Clin Perinatol.

[27] Chehade H, Simeoni U, Guignard JP, Boubred F (2018). Preterm birth: long term cardiovascular and renal consequences. Curr Pediatr Rev.

[28] McCarthy EK, Dempsey EM, Kiely ME (2019). Iron supplementation in preterm and low-birth-weight infants: a systematic review of intervention studies. Nutr Rev.

[29] Burris AD, Burris J, Järvinen KM (2020). Cow's Milk protein allergy in term and preterm infants: clinical manifestations, immunologic pathophysiology, and management strategies. Neoreviews..

[30] Abrams SA (2020). Vitamin D in Preterm and Full-Term Infants. Ann Nutr Metab.

[31] Schwartz E, Zelig R, Parker A, Johnson S (2017). Vitamin a supplementation for the prevention of Bronchopulmonary dysplasia in preterm infants: an update. Nutr Clin Pract.

[32] Ghomi H, Yadegari F, Soleimani F, Knoll BL, Noroozi M, Mazouri A (2019). The effects of premature infant oral motor intervention (PIOMI) on oral feeding of preterm infants: a randomized clinical trial. Int J Pediatr Otorhinolaryngol.

[33] Spillane A, Belton S, McDermott C, Issartel J, Osborne RH, Elmer S, Murrin C (2020). Development and validity testing of the adolescent health literacy questionnaire (AHLQ): protocol for a mixed methods study within the Irish school setting. BMJ Open.

[34] Chmura-Kraemer H, Periyakoil VS, Noda A (2002). Kappa coefficients in medical research. Stat Med.

[35] Gerasimidis K, Keane O, Macleod I, Flynn DM, Wright CM (2010). A four-stage evaluation of the Paediatric Yorkhill malnutrition score in a tertiary paediatric hospital and a district general hospital. Br J Nutr.

[36] Hai Y, Qin G (2020). Direct estimation of the area under the receiver operating characteristic curve with verification biased data. Stat Med.

[37] Lee YJ, Yang HR (2019). Comparison of four nutritional screening tools for Korean hospitalized children. Nutr Res Pract.

[38] Ong SH, Chee WSS, Lapchmanan LM, Ong SN, Lua ZC, Yeo JX (2019). Validation of the subjective global nutrition assessment (SGNA) and screening tool for the assessment of malnutrition in Paediatrics (STAMP) to identify malnutrition in hospitalized Malaysian children. J Trop Pediatr.

[39] Barrett BJ, Fardy JM (2021). Evaluation of diagnostic tests. Methods Mol Biol.

[40] Schober P, Boer C, Schwarte LA (2018). Correlation coefficients: appropriate use and interpretation. Anesth Analg.

[41] Maciel JRV, Nakano EY, Carvalho KMB, Dutra ES (2020). STRONGkids validation: tool accuracy. J Pediatr.

[42] Pimenta FS, Oliveira CM, Hattori WT, Teixeira KR (2018). Agreement between subjective global nutritional assessment and the nutritional assessment of the World Health Organization. J Pediatr.

[43] White M, Lawson K, Ramsey R, Dennis N, Hutchinson Z, Soh XY, Matsuyama M, Doolan A, Todd A, Elliott A, Bell K, Littlewood R (2016). Simple nutrition screening tool for pediatric inpatients. JPEN J Parenter Enteral Nutr.

[44] Ipsen J, Nowak-Szczepanska N, Gomula A, Aßmann C, Hermanussen M (2016). The association of body height, height variability and inequality. Anthropol Anz.

[45] Bach CC, Henriksen TB, Larsen RT, Aagaard K, Matthiesen NB (2020). Head circumference at birth and school performance: a nationwide cohort study of 536,921 children. Pediatr Res.

[46] Catena A, Martínez-Zaldívar C, Diaz-Piedra C, Torres-Espínola FJ, Brandi P, Pérez-García M, Decsi T, Koletzko B, Campoy C (2019). On the relationship between head circumference, brain size, prenatal long-chain PUFA/5-methyltetrahydrofolate supplementation and cognitive abilities during childhood. Br J Nutr.

