# Supplementary material for: Development of a nutritional risk screening tool for preterm children in outpatient settings during a complementary feeding period: a pilot study
Source: BMC Pediatr. 2022 Dec 7;22:702. doi: 10.1186/s12887-022-03774-5 (PMC9730637; doi:10.1186/s12887-022-03774-5)
Supplement: Supplementary file 2 — Additional file 2: Appendix 2. Nutritional Risk Screening Tool for Preterm Infants at the Corrected Age of 8–11 Months. [file 12887_2022_3774_MOESM2_ESM.docx]

Appendix 2 Nutritional Risk Screening Tool for Preterm Infants at the Corrected Age of 8-11 Months

| **1.Health Status** |
| --- |
| 1.1 Current diseases  A None (Score 0)  B Gastrointestinal disorders/ Cardiopulmonary disorders/ Neurological disorders/ Metabolic disorders/ Haematological system diseases with functional impairment (Score 2)  C Acute diseases/ Allergic diseases (Score 1)  D More than one of the above diseases (Score 3) |
|  |
| **2.Feeding Practices** |
| 2.1 Milk intake per 24 hours (please mind if the formula was prepared as instructed.)  A <600ml/d (Score 2) B 600-800ml/d (Score 0) C >800ml/d (Score -1) |
| 2.2 Nutritional fortifierS use (Please mind if the fortifier was used as instructed.)  A More than half of the milk intake per day (Score -2) B Less than half of the milk intake per day (Score -1) C None (Score 0) |
| 2.3 Special formula use  A More than half of the milk intake per day (Score 2) B Less than half of the milk intake per day (Score 1) C None (Score 0) |
| 2.4 Frequency of different kinds of food intake (at least 10 grams of the kind of food per day)  A Red meat, 4 to 7 days per week (Score 0), 1 to 3 days per week (Score 0.5), 1 to 2 days per months (Score 1), None (Score 2)  B White meat (Score 0)  C Animal viscus, 6 to 7 days per week (Score -1), 4 to 5 days per week (Score -0.5), 1 to 3 days per week (Score 0), 1 to 2 days per months (Score 0.5), None (Score 1)  D Egg and yolk, 4 to 7 days per week (Score 0), 1 to 3 days per week (Score 0.5), 1 to 2 days per months (Score 1), None (Score 2)  E Vegetables and fruits (Score 0)  F Soybean products (Score 0) |
| 2.5 Cereal intake per day  A <25g/d (Score 1) B 25 to 75g/d (Score 0) C >75g/d (Score 1) |
| 2.6 Animal food intake per day  A None (Score 2) B <25g/d (Score 1) C ≥25g/d (Score 0) |
| 2.7 Energy density of complementary food  A Complementary food in liquid form (Score 1) B Complementary food in semi-solid form (Score 0) |
| 2.8 Perceived eating difficulty  A Easy (Score 0) B Difficult (Score 1) C Very difficult (Score 2) |
|  |
| **3.Nutrient Supplementation** |
| 3.1 Supply Vitamin D 400 to 800 international units as daily dose  A 6 to 7 days per week (Score 0) B 4 to 5 days per week (Score 0.5) C 1 to 3 days per week (Score 1) D None (Score 2) |
| 3.2 Hours spent outdoors per week  A More than 7 hours (Score -1) B 5 to 7 hours (Score 0) C 3 to 5 hours (Score 0.5) D 1 to 3 hours (Score 1) E Less than 1 hours (Score 2) |
| 3.3 Supply vitamin A 1333 to 1500 international units as daily dose  A 4 to 7 days per week (Score 0) B 1 to 3 days per week (Score 0.5) C None (Score 1) |
| 3.4 Supply iron element two milligram per kilogram of weight as daily dose  A 6 to 7 days per week (Score -1) B 4 to 5 days per week (Score 0) C 1 to 3 days per week (Score 0.5) D None (Score 1) |
| 3.5 Other nutrients such as calcium, zinc, multivitamins, DHA, prebiotics  A None (Score 0) B Yes (score -1 with calcium supplement) |
|  |
| **4. Anthropometric Assessment** |
| 4.1 Z-score of birth weight  A ≥-1 (Score 0) B -1~-2 (Score 1) C <-2 (Score 2) |
| 4.2 Z-score of birth length  A ≥-1 (Score 0) B -1~-2 (Score 1) C <-2 (Score 2) |
| 4.3 Z-score of birth head circumference  A ≥-1 (Score 0) B -1~-2 (Score 1) C <-2 (Score 2) |
| 4.4 Decrease in Z-score of body weight (poor weight gain, z-score on the interview day minus the z-score from last time < -0.2)  A No (Score 0) B Yes (Score 2) |
| 4.5 Decrease in Z-score of body length (poor body length growth)  A No (Score 0) B Yes (Score 2) |
| 4.6 Decrease in Z-score of head circumference (poor head circumference growth)  A No (Score 0) B Yes (Score 2) |
