# Supplementary material for: Development of a nutritional risk screening tool for preterm children in outpatient settings during a complementary feeding period: a pilot study
Source: BMC Pediatr. 2022 Dec 7;22:702. doi: 10.1186/s12887-022-03774-5 (PMC9730637; doi:10.1186/s12887-022-03774-5)
Supplement: Supplementary file 4 — Additional file 4: Appendix 4–1. Univariate analysis of responses to the screening tool and the z-scores classification of body weight, length and head circumference for preterm infants at the corrected age of 5–7 months [n (%)]. Appendix 4–2. Binary logistic regression analysis of responses to the screening tool and the z-scores classification of body weight, length and head circumference for preterm infants at the corrected age of 5–7 months (P > 0.900 were not shown). [file 12887_2022_3774_MOESM4_ESM.docx]

Appendix4-1 Univariate analysis of responses to the screening tool and the z-scores classification of body weight, length and head circumference for preterm infants at the corrected age of 5-7 months [n (%)]

|  | **WTZ≥-2** | **WTZ<-2** | **P** | **LGZ≥-2** | **LGZ<-2** | **P** | **HCZ≥-2** | **HCZ<-2** | **P** |
| --- | --- | --- | --- | --- | --- | --- | --- | --- | --- |
| n | 97 | 7 |  | 96 | 8 |  | 98 | 6 |  |
| BWTZ≥-1 | 82(84.54) | 4(57.14) | 0.009 | 83(86.46) | 3(37.5) | <0.001 | 83(84.69) | 3(50.00) | 0.003 |
| BWTZ-1~-2 | 12(12.37) | 1(14.28) |  | 13(13.54) | 0 |  | 12(12.24) | 1(16.67) |  |
| BWTZ<-2 | 3(3.09) | 2(28.57) |  | 0 | 5(62.5) |  | 3(3.06) | 2(33.33) |  |
| BLGZ≥-1 | 67(69.07) | 5(71.43) | <0.001 | 68(70.83) | 4(50.00) | <0.001 | 68(69.39) | 4(66.67) | 0.917 |
| BLGZ-1~-2 | 30(30.93) | 0 |  | 28(29.17) | 2(25.00) |  | 28(28.57) | 2(33.33) |  |
| BLGZ<-2 | 0 | 2(28.57) |  | 0 | 2(25.00) |  | 2(2.04) | 0 |  |
| BHCZ≥-1 | 74(76.29) | 3(42.85) | 0.083 | 74(77.08) | 3(37.50) | <0.001 | 75(76.53) | 2(33.33) | 0.033 |
| BHCZ-1~-2 | 16(16.49) | 2(28.57) |  | 17(17.71) | 1(12.50) |  | 16(16.32) | 2(33.33) |  |
| BHCZ<-2 | 7(7.22) | 2(28.57) |  | 5(5.21) | 4(50.00) |  | 7(7.14) | 2(33.33) |  |
| Current diseases |  |  | 0.867 |  |  | 0.932 |  |  | 0.894 |
| None | 82(84.54) | 7(100.00) |  | 82(85.42) | 7(87.50) |  | 84(85.71) | 5(83.33) |  |
| Neurological disorders | 2(2.06) | 0 |  | 2(2.08) | 0 |  | 2(2.04) | 0 |  |
| Haematological system diseases | 2(2.06) | 0 |  | 2(2.08) | 0 |  | 2(2.04) | 0 |  |
| Acute diseases | 3(3.09) | 0 |  | 3(3.12) | 0 |  | 3(3.06) | 0 |  |
| Allergic diseases | 8(8.25) | 0 |  | 7(7.29) | 1(12.50) |  | 7(7.14) | 1(16.66) |  |
| Milk intake |  |  | 0.305 |  |  | 0.151 |  |  | 0.080 |
| <600ml/d | 17(17.52) | 2(28.57) |  | 16(16.67) | 3(37.50) |  | 16(16.32) | 3(50.00) |  |
| 600-800ml/d | 56(57.73) | 5(71.43) |  | 56(58.33) | 5(62.50) |  | 58(59.18) | 3(50.00) |  |
| >800ml/d | 24(24.74) | 0 |  | 24(25.00) | 0 |  | 24(24.49) | 0 |  |
| Nutritional fortifier usage |  |  | <0.001 |  |  | <0.001 |  |  | 0.577 |
| None | 92(94.84) | 3(42.86) |  | 93(96.87) | 2(25.00) |  | 90(91.84) | 5(83.33) |  |
| <1/2 milk intake | 2(2.06) | 0 |  | 2(2.08) | 0 |  | 2(2.04) | 0 |  |
| ≥1/2 milk intake | 3(3.09) | 4(57.14) |  | 1(1.04) | 6(75.00) |  | 6(6.12) | 1(16.66) |  |
| Special Formula |  |  | 0.503 |  |  | 0.144 |  |  | 0.902 |
| None | 81(83.51) | 5(71.43) |  | 81(84.37) | 5(62.50) |  | 81(82.65) | 5(83.33) |  |
| <1/2 milk intake | 3(3.09) | 0 |  | 2(2.08) | 1(12.50) |  | 3(3.06) | 0 |  |
| ≥1/2 milk intake | 13(13.40) | 2(28.57) |  | 13(13.54) | 2(25.00) |  | 14(14.28) | 1(16.66) |  |
| Cereal intake | 61(62.89) | 5(71.43) | 0.650 | 62(64.58) | 4(50.00) | 0.459 | 61(62.24) | 5(83.33) | 0.298 |
| Animal food intake | 20(20.62) | 0 | 0.341 | 19(19.79) | 1(12.50) | 0.615 | 17(17.35) | 3(50.00) | 0.084 |
| Enough energy density | 83(85.57) | 3(42.85) | 0.016 | 84(87.50) | 2(25.00) | <0.001 | 81(82.65) | 5(83.33) | 0.301 |
| Perceived eating difficulty |  |  | 0.465 |  |  | 0.159 |  |  | 0.325 |
| Easy | 80(82.47) | 5(71.43) |  | 80(83.33) | 5(62.50) |  | 81(82.65) | 4(66.67) |  |
| Difficult | 17(17.53) | 2(21.87) |  | 16(16.67) | 3(37.50) |  | 17(17.35) | 2(33.33) |  |
| Very difficult | 0 | 0 |  | 0 | 0 |  | 0 | 0 |  |
| Vitamin D supplement (400-800IU/d) |  |  | 0.910 |  |  | 0.370 |  |  | 0.928 |
| None | 3(3.09) | 0 |  | 2(2.08) | 1(12.50) |  | 3(3.06) | 0 |  |
| 1-3 days per week | 2(2.06) | 0 |  | 2(2.08) | 0 |  | 2(2.04) | 0 |  |
| 4-5 days per week | 2(2.06) | 0 |  | 2(2.08) | 0 |  | 2(2.04) | 0 |  |
| 6-7 days per week | 90(92.78) | 7(100.00) |  | 90(93.75) | 7(87.50) |  | 91(92.85) | 6(100.00) |  |
| Hours spent outdoors per week |  |  | 0.210 |  |  | 0.100 |  |  | 0.003 |
| <1 hour | 27(27.83) | 0 |  | 27(28.12) | 0 |  | 25(25.51) | 2(33.33) |  |
| 1-3 hours | 20(20.62) | 3(42.86) |  | 19(19.79) | 4(50.00) |  | 22(22.45) | 1(16.67) |  |
| 3-5 hours | 40(41.24) | 4(57.14) |  | 40(41.67) | 4(50.00) |  | 44(44.90) | 0 |  |
| 5-7 hours | 10(10.31) | 0 |  | 10(10.42) | 0 |  | 7(7.14) | 3(50.00) |  |
| >7 hours | 0 | 0 |  | 0 | 0 |  | 0 | 0 |  |
| Vitamin A supplement (1333-1500IU/d) |  |  | 0.162 |  |  | 0.498 |  |  | 0.213 |
| None | 20(20.62) | 0 |  | 18(18.75) | 2(25.00) |  | 20(20.40) | 0 |  |
| 1-3 days per week | 14(14.43) | 0 |  | 14(14.58) | 0 |  | 12(12.24) | 2(33.33) |  |
| 4-5 days per week | 0 | 0 |  | 0 | 0 |  | 0 | 0 |  |
| 6-7 days per week | 63(64.95) | 7(100.00) |  | 64(66.67) | 6(75.00) |  | 66(67.34) | 4(66.67) |  |
| Iron supplement (2mg/kg/d) |  |  | 0.589 |  |  | 0.965 |  |  | 0.295 |
| None | 50(51.54) | 2(28.57) |  | 48(50.00) | 4(50.00) |  | 50(51.02) | 2(33.33) |  |
| 1-3 days per week | 14(14.43) | 2(28.57) |  | 15(15.62) | 1(12.50) |  | 16((16.32) | 0 |  |
| 4-5 days per week | 2(2.06) | 0 |  | 2(2.08) | 0 |  | 2(2.04) | 0 |  |
| 6-7 days per week | 31(31.96) | 3(42.86) |  | 31(32.29) | 3(37.50) |  | 30(30.61) | 4(66.67) |  |
| Calcium supplement | 45(46.39) | 4(57.14) | 0.704 | 47(48.96) | 2(25.00) | 0.159 | 48(48.98) | 1(16.67) | 0.210 |
| Zinc supplement | 1(1.03) | 0 | 1.000 | 1(1.04) | 0 | 1.000 | 1(1.02) | 0 | 1.000 |
| Poor weight gain | 17(17.53) | 3(42.86) | 0.128 | 17(17.71) | 3(37.50) | 0.179 | 19(19.39) | 1(16.67) | 1.000 |
| Poor body length growth | 22(22.68) | 2(28.57) | 0.661 | 23(23.96) | 1(12.50) | 0.678 | 23(23.47) | 1(16.67) | 1.000 |
| Poor head circumference growth | 36(37.11) | 3(42.86) | 1.000 | 38(39.58) | 1(12.50) | 0.253 | 38(38.77) | 1(16.67) | 0.406 |

WT/LG/HCZ: z-scores of body weight/length/head circumference one month after the interview; BWT/LG/HCZ: z-scores of birth weight/length/head circumference; all were analyzed by chi-square test or Fisher`s exact test.

Appendix4-2 Binary logistic regression analysis of responses to the screening tool and the z-scores classification of body weight, length and head circumference for preterm infants at the corrected age of 5-7 months (P > 0.900 were not shown)

|  | **OR(95%CI)** | **P** |
| --- | --- | --- |
| **Model to predict underweight** |  |  |
| Z-score of birthweight ≥-1 | 0.455(0.024-8.819) | 0.603 |
| Z-score of birthweight -1~-2 | 0.049(0.001-1.874) | 0.105 |
| Z-score of birthweight <-2 (reference) | - | 0.244 |
| Nutritional fortifier usage |  |  |
| None | 0.009(0.000-0.230) | 0.004 |
| <1/2 milk intake | - | - |
| ≥1/2 milk intake (reference) | - | 0.017 |
| Cereal intake | 0.229(0.016-3.361) | 0.282 |
| Animal food intake | 0.512(0.038-6.864) | 0.613 |
| Enough energy density | 0.262(0.019-3.639) | 0.318 |
| Poor weight gain | 0.103(0.012-0.867) | 0.037 |
| **Model to predict stunting** |  |  |
| Z-score of birth weight ≥-1 | 0.233(0.011-5.107) | 0.355 |
| Z-score of birth weight -1~-2 | 0.073(0.004-1.519) | 0.091 |
| Z-score of birth weight <-2 (reference) | - | 0.083 |
| Z-score of birth length ≥-1 | 0.120(0.009-1.526) | 0.102 |
| Z-score of birth length -1~-2 | 0.429(0.030-6.105) | 0.533 |
| Z-score of birth length <-2 (reference) | - | 0.027 |
| Milk intake |  |  |
| <600ml/d | 10.264(0.993-106.045) | 0.051 |
| 600-800ml/d | 8.758(1.006-76.237) | 0.049 |
| >800ml/d (reference) | - | 0.126 |
| Nutritional fortifier usage |  |  |
| None | 0.178(0.034-0.943) | 0.042 |
| <1/2 milk intake | - | - |
| ≥1/2 milk intake (reference) | - | 0.127 |
| Animal food intake | 0.533(0.130-2.181) | 0.381 |
| Vitamin D supplement (400-800IU/d) |  |  |
| None | 0.358(0.005-24.761) | 0.634 |
| 1-3 days per week | - | - |
| 4-5 days per week | 0.271(0.000-257.597) | 0.709 |
| 6-7 days per week (reference) | - | 0.951 |
| Vitamin A supplement (1333-1500IU/d) |  |  |
| None | 1.249(0.339-4.599) | 0.738 |
| 1-3 days per week | 0.785(0.065-9.536) | 0.849 |
| 4-5 days per week | 9.622(0.009-10173.03) | 0.524 |
| 6-7 days per week (reference) | - | 0.909 |
| Calcium supplement | 0.597(0.209-1.704) | 0.335 |
| Poor body length growth | 0.085(0.002-3.579) | 0.197 |
| **Model to predict microcephaly** |  |  |
| Z-score of birth weight ≥-1 | 2.524(0.000-13822.280) | 0.833 |
| Z-score of birth weight -1~-2 | 0.500(0.002-129.905) | 0.807 |
| Z-score of birth weight <-2 (reference) | - | 0.728 |
| Z-score of birth head circumference ≥-1 | 0.258(0.003-20.664) | 0.545 |
| Z-score of birth head circumference -1~-2 | 0.086(0.001-10.858) | 0.320 |
| Z-score of birth head circumference <-2 (reference) | - | 0.598 |
| Milk intake |  |  |
| <600ml/d | 3.709(0.107-128.850) | 0.469 |
| 600-800ml/d | 0.652(0.033-12.889) | 0.779 |
| >800ml/d (reference) | - | 0.504 |
| Nutritional fortifier usage |  |  |
| None | 0.016(0.001-0.468) | 0.016 |
| <1/2 milk intake | - | - |
| ≥1/2 milk intake (reference) | - | 0.056 |
| Animal food intake | 0.093(0.006-1.430) | 0.088 |
| Hours spent outdoors per week |  |  |
| <1hour | 0.061(0.001-5.648) | 0.226 |
| 1-3 hours | 0.287(0.014-5.922) | 0.419 |
| 3-5 hours | - | 0.687 |
| 5-7 hours (reference) | - | - |
| >7 hours | - | - |
| Poor head circumference growth | 1.612(0.108-24.169) | 0.730 |

1. Model to predict underweight included factors of z-scores of birth weight/length/head circumference, nutritional fortifier usage, milk/cereal/animal food intake, food energy density, perceived eating difficulty, poor weight gain.

2. Model to predict stunting included factors of z-scores of birth weight/length/head circumference, nutritional fortifier usage, milk/animal food intake, food energy density, vitamin D and A supplement, calcium supplement, hours spent outdoors per week, poor body length growth.

3. Model to predict microcephaly included factors of z-scores of birth weight/head circumference, nutritional fortifier usage, milk/animal food intake, vitamin D supplement, hours spent outdoors per week, poor head circumference growth.
