# Supplementary material for: Development of a nutritional risk screening tool for preterm children in outpatient settings during a complementary feeding period: a pilot study
Source: BMC Pediatr. 2022 Dec 7;22:702. doi: 10.1186/s12887-022-03774-5 (PMC9730637; doi:10.1186/s12887-022-03774-5)
Supplement: Supplementary file 5 — Additional file 5: Appendix 5–1. Univariate analysis of responses to the screening tool and the z-scores classification of body weight, length and head circumference for preterm infants at the corrected age of 8–11 months [n (%)]. Appendix 5–2. Binary logistic regression analysis of responses to the screening tool and the z-scores classification of body weight, length and head circumference for preterm infants at the corrected age of 8–11 months (P > 0.900 were not shown). [file 12887_2022_3774_MOESM5_ESM.docx]

Appendix5-1 Univariate analysis of responses to the screening tool and the z-scores classification of body weight, length and head circumference for preterm infants at the corrected age of 8-11 months [n (%)]

|  | **WTZ≥-2** | **WTZ<-2** | **P** | **LGZ≥-2** | **LGZ<-2** | **P** | **HCZ≥-2** | **HCZ<-2** | **P** |
| --- | --- | --- | --- | --- | --- | --- | --- | --- | --- |
| n | 99 | 11 |  | 105 | 5 |  | 104 | 6 |  |
| BWTZ≥-1 | 83(83.84) | 3(27.27) | <0.001 | 86(81.90) | 0 | <0.001 | 85(81.73) | 1(16.67) | <0.001 |
| BWTZ-1~-2 | 15(15.15) | 3(27.27) |  | 16(15.24) | 2(40.00) |  | 16(15.38) | 2(33.33) |  |
| BWTZ<-2 | 1(1.01) | 5(45.45) |  | 3(2.86) | 3(60.00) |  | 3(2.88) | 350.00) |  |
| BLGZ≥-1 | 83(83.84) | 5(45.45) | <0.001 | 86(81.90) | 2(40.00) | <0.001 | 85(81.73) | 3(50.00) | 0.001 |
| BLGZ-1~-2 | 12(12.12) | 0 |  | 12(11.43) | 0 |  | 12(11.54) | 0 |  |
| BLGZ<-2 | 4(4.04) | 6(54.54) |  | 7(6.67) | 3(60.00) |  | 7(6.73) | 3(50.00) |  |
| BHCZ≥-1 | 72(72.72) | 4(36.36) | 0.002 | 76(72.38) | 0 | 0.001 | 75(72.11) | 1(16.67) | 0.006 |
| BHCZ-1~-2 | 18(18.18) | 2(18.18) |  | 18(17.14) | 2(40.00) |  | 18(17.31) | 2(33.33) |  |
| BHCZ<-2 | 9(9.09) | 5(45.45) |  | 11(10.47) | 3(60.00) |  | 11(10.58) | 3(50.00) |  |
| Current diseases |  |  | 0.353 |  |  | 0.640 |  |  | 0.583 |
| None | 83(83.84) | 11(100.00) |  | 89(84.76) | 5(100.00) |  | 88(84.62) | 6(100.00) |  |
| Neurological disorders | 1(1.01) | 0 |  | 1(0.95) | 0 |  | 1(0.96) | 0 |  |
| Allergic diseases/Acute diseases | 15(15.15) | 0 |  | 15(14.28) | 0 |  | 15(14.42) | 0 |  |
| Milk intake |  |  | 0.129 |  |  | 0.305 |  |  | 0.001 |
| <600ml/d | 28(28.28) | 6(54.54) |  | 31(29.52) | 3(60.00) |  | 28(26.92) | 6(100.00) |  |
| 600-800ml/d | 56(56.56) | 5(45.45) |  | 59(56.19) | 2(40.00) |  | 61(58.65) | 0 |  |
| >800ml/d | 15(15.15) | 0 |  | 15(14.28) | 0 |  | 15(14.42) | 0 |  |
| Nutritional fortifier usage |  |  | 0.003 |  |  | <0.001 |  |  | 0.008 |
| None | 93(93.94) | 8(72.73) |  | 99(94.28) | 2(40.00) |  | 97(93.27) | 4(66.67) |  |
| <1/2 milk intake | 3(3.03) | 0 |  | 3(2.86) | 0 |  | 3(2.88) | 0 |  |
| ≥1/2 milk intake | 3(3.03) | 3(27.27) |  | 3(2.86) | 3(60.00) |  | 4(3.85) | 2(33.33) |  |
| Special Formula |  |  | 0.703 |  |  | 0.860 |  |  | 0.833 |
| None | 93(93.94) | 11(100.00) |  | 99(94.28) | 5(100.00) |  | 98(94.23) | 6(100.00) |  |
| <1/2 milk intake | 1(1.01) | 0 |  | 1(0.95) | 0 |  | 1(0.96) | 0 |  |
| ≥1/2 milk intake | 5(5.05)) | 0 |  | 5(4.76) | 0 |  | 5(4.81) | 0 |  |
| Red meat intake frequency |  |  | 0.112 |  |  | 0.458 |  |  | 0.267 |
| None | 13(13.13) | 0 |  | 13(12.38) | 0 |  | 13(12.50) | 0 |  |
| 1-3 days per week | 22(22.22) | 0 |  | 22(20.95) | 0 |  | 22(21.15) | 0 |  |
| 4-5 days per week | 42(42.42) | 8(72.72) |  | 47(44.76) | 3(60.00) |  | 47(45.19) | 3(50.00) |  |
| 6-7 days per week | 22(22.22) | 3(27.27) |  | 23(21.90) | 2(40.00) |  | 22(21.15) | 3(50.00) |  |
| White meat intake frequency |  |  | 0.086 |  |  | 0.421 |  |  | 0.456 |
| None | 33(33.33) | 2(18.18) |  | 35(33.33) | 0 |  | 34(32.69) | 1(16.67) |  |
| 1-3 days per week | 43(43.43) | 3(27.27) |  | 43(40.95) | 3(60.00) |  | 44(42.31) | 2(33.33) |  |
| 4-5 days per week | 20(20.20) | 6(54.54) |  | 24(22.86) | 2(40.00) |  | 23(22.11) | 3(50.00) |  |
| 6-7 days per week | 3(3.03) | 0 |  | 3(2.86) | 0 |  | 3(2.88) | 0 |  |
| Animal viscus intake frequency |  |  | 0.496 |  |  | 0.500 |  |  | <0.001 |
| None | 65(65.65) | 5(45.45) |  | 68(64.76) | 2(40.00) |  | 70(67.31) | 0 |  |
| 1-2 days per month | 4(4.04) | 1(9.09) |  | 5(4.76) | 0 |  | 3(2.88) | 2(33.33) |  |
| 1-3 days per week | 28(28.28) | 5(45.45) |  | 30(28.57) | 3(60.00) |  | 29(27.88) | 4(66.67) |  |
| 4-5 days per week | 2(2.02) | 0 |  | 2(1.90) | 0 |  | 2(1.92) | 0 |  |
| 6-7 days per week | 0 | 0 |  | 0 | 0 |  | 0 | 0 |  |
| Egg and yolk intake frequency |  |  | 0.917 |  |  | 0.121 |  |  | 0.305 |
| None | 16(16.16) | 2(18.18) |  | 16(15.24) | 2(40.00) |  | 18(17.31) | 0 |  |
| 1-3 days per week | 32(32.32) | 3(27.27) |  | 35(33.33) | 0 |  | 32(30.77) | 3(50.00) |  |
| 4-5 days per week | 32(32.32) | 3(27.27) |  | 32(30.48) | 3(60.00) |  | 32(30.77) | 3(50.00) |  |
| 6-7 days per week | 19(19.19) | 3(27.27) |  | 22(20.95) | 0 |  | 22(21.15) | 0 |  |
| Cereal intake |  |  | 0.583 |  |  | 0.713 |  |  | 0.154 |
| <25g/d | 40(40.40) | 3(27.27) |  | 41(39.05) | 2(40.00) |  | 42(40.38) | 1(16.67) |  |
| 25-50g/d | 49(49.49) | 6(54.54) |  | 52(49.52) | 3(60.00) |  | 52(50.00) | 3(50.00) |  |
| 50-75g/d | 10(10.10) | 2(18.18) |  | 12(11.43) | 0 |  | 10(9.61) | 2(33.33) |  |
| Animal food intake |  |  | 0.558 |  |  | 0.926 |  |  | 0.845 |
| None | 7(7.07) | 0 |  | 7(6.67) | 0 |  | 7(6.73) | 0 |  |
| <25g/d | 52(52.52) | 8(72.72) |  | 57(54.28) | 3(60.00) |  | 57(54.81) | 3(50.00) |  |
| 25-50g/d | 38(38.38) | 3(27.27) |  | 39(37.14) | 2(40.00) |  | 38(36.54) | 3(50.00) |  |
| 50-75g/d | 2(2.02) | 0 |  | 2(1.90) | 0 |  | 2(1.92) | 0 |  |
| Enough energy density | 97(97.98) | 11(100.00) | 1.000 | 103(98.09) | 5(100.00) | 1.000 | 102(98.08) | 6(100.00) | 1.000 |
| Perceived eating difficulty |  |  | 0.004 |  |  | 0.324 |  |  | 0.380 |
| Easy | 72(72.73) | 3(27.27) |  | 73(69.52) | 2(40.00) |  | 72(69.23) | 3(50.00) |  |
| Difficult | 27(27.27) | 8(72.73) |  | 32(30.47) | 3(60.00) |  | 32(30.77) | 3(50.00) |  |
| Very difficult | 0 | 0 |  | 0 | 0 |  | 0 | 0 |  |
| Vitamin D supplement (400-800IU/d) |  |  | 0.651 |  |  | 0.873 |  |  | 0.837 |
| None | 1(1.01) | 0 |  | 1(0.95) | 0 |  | 1(0.96) | 0 |  |
| 1-3 days per week | 6(6.06) | 0 |  | 6(5.71) | 0 |  | 6(5.77) | 0 |  |
| 4-5 days per week | 6(6.06) | 0 |  | 6(5.71) | 0 |  | 6(5.77) | 0 |  |
| 6-7 days per week | 86(86.87) | 11(100.00) |  | 92(87.62) | 5(100.00) |  | 91(87.50) | 6(100.00) |  |
| Hours spent outdoors per week |  |  | 0.311 |  |  | 0.064 |  |  | 0.199 |
| <1 hours | 19(19.19) | 3(27.27) |  | 19(18.09) | 3(60.00) |  | 19(18.27) | 3(50.00) |  |
| 1-3 hours | 25(25.25) | 1(9.09) |  | 26(24.76) | 0 |  | 26(25.00) | 0 |  |
| 3-5 hours | 30(30.30) | 2(18.18) |  | 32(30.48) | 0 |  | 30(28.85) | 2(33.33) |  |
| 5-7 hours | 25(25.25) | 5(45.45) |  | 28(26.67) | 2(40.00) |  | 29(27.88) | 1(16.67) |  |
| >7 hours | 0 | 0 |  | 0 | 0 |  | 0 | 0 |  |
| Vitamin A supplement (1333-1500IU/d) |  |  | 0.802 |  |  | 0.884 |  |  | 0.605 |
| None | 27(27.27) | 3(27.27) |  | 28(26.67) | 2(40.00) |  | 27(25.96) | 3(50.00) |  |
| 1-3 days per week | 19(19.19) | 3(27.27) |  | 21(20.00) | 1(20.00) |  | 21(20.19) | 1(16.67) |  |
| 4-5 days per week | 6(6.06) | 0 |  | 6(5.71) | 0 |  | 6(5.77) | 0 |  |
| 6-7 days per week | 47(47.47) | 5(45.45) |  | 50(47.62) | 2(40.00) |  | 50(48.08) | 2(33.33) |  |
| Iron supplement (2mg/kg/d) |  |  | 0.847 |  |  | 0.209 |  |  | 0.265 |
| None | 59(59.59) | 6(54.54) |  | 62(59.05) | 3(60.00) |  | 61(58.65) | 4(66.67) |  |
| 1-3 days per week | 24(24.24) | 3(27.27) |  | 27(25.71) | 0 |  | 27(25.96) | 0 |  |
| 4-5 days per week | 4(4.04) | 0 |  | 4(3.81) | 0 |  | 4(3.85) | 0 |  |
| 6-7 days per week | 12(12.12) | 2(18.18) |  | 12(11.43) | 2(40.00) |  | 12(11.54) | 2(33.33) |  |
| Calcium supplement | 61(61.62) | 6(54.54) | 0.748 | 65(61.90) | 2(40.00) | 0.377 | 64(61.54) | 3(50.00) | 0.677 |
| Zinc supplement | 3(3.03) | 3(27.27) | 0.013 | 4(3.81) | 2(40.00) | 0.023 | 3(2.88) | 3(50.00) | 0.002 |
| Poor weight gain | 38(38.38) | 3(27.27) | 0.533 | 39(37.14) | 2(40.00) | 1.000 | 38(36.54) | 3(50.00) | 0.669 |
| Poor body length growth | 32(32.32) | 6(54.54) | 0.184 | 33(31.43) | 5(100.00) | 0.004 | 34(32.69) | 4(66.67) | 0.179 |
| Poor head circumference growth | 31(31.31) | 2(18.18) | 0.500 | 31(29.52) | 2(40.00) | 0.635 | 31(29.81) | 2(33.33) | 1.000 |

WT/LG/HCZ: z-scores of body weight/length/head circumference one month after the interview; BWT/LG/HCZ: z-scores of birth weight/length/head circumference; all were analyzed by chi-square test or Fisher`s exact test.

Appendix5-2 Binary logistic regression analysis of responses to the screening tool and the z-scores classification of body weight, length and head circumference for preterm infants at the corrected age of 8-11 months (P > 0.900 were not shown).

|  | **OR(95%CI)** | **P** |
| --- | --- | --- |
| **Model to predict underweight** |  |  |
| Z-score of birth weight ≥-1 | 0.014(0.001-0.209) | 0.002 |
| Z-score of birth weight -1~-2 | 0.048(0.002-1.040) | 0.053 |
| Z-score of birth weight <-2 (reference) | - | 0.008 |
| Z-score of birth length ≥-1 | 0.043(0.003-0.654) | 0.024 |
| Z-score of birth length -1~-2 | - | - |
| Z-score of birth length <-2 (reference) | - | 0.077 |
| Nutritional fortifier usage |  |  |
| None | 0.081(0.005-1.398) | 0.084 |
| <1/2 milk intake | - | - |
| ≥1/2 milk intake (reference) | - | 0.224 |
| Cereal intake |  |  |
| <25g/d | 0.293(0.014-6.331) | 0.434 |
| 25-50g/d | 0.439(0.028-6.798) | 0.556 |
| 50-75g/d (reference) | - | 0.736 |
| Perceived eating difficulty |  |  |
| Easy | 0.147(0.026-0.826) | 0.029 |
| Difficult (reference) | - | - |
| Very difficult | - | - |
| Poor weight gain | 5.010(0.421-59.616) | 0.202 |
| **Model to predict stunting** |  |  |
| Z-score of birth weight ≥-1 | - | - |
| Z-score of birth weight -1~-2 | 0.091(0.005-1.547) | 0.097 |
| Z-score of birth weight <-2 (reference) | - | 0.253 |
| **Model to predict microcephaly** |  |  |
| Z-score of birth head circumference ≥-1 | 0.036(0.002-0.828) | 0.038 |
| Z-score of birth head circumference -1~-2 | 0.250(0.008-7.452) | 0.423 |
| Z-score of birth head circumference <-2 (reference) | - | 0.107 |

1. Model to predict underweight included factors of z-scores of birth weight/length/head circumference, nutritional fortifier usage, frequency of white meat intake, milk/cereal/animal food intake, food energy density, perceived eating difficulty, poor weight gain.

2. Model to predict stunting included factors of z-scores of birth weight/length/head circumference, nutritional fortifier usage, milk/animal food intake, vitamin D and A supplement, calcium supplement, hours spent outdoors per week, poor body length growth.

3. Model to predict microcephaly included factors of z-scores of birth weight/length/head circumference, nutritional fortifier usage, milk/animal food intake, frequency of red meat/animal viscus/egg and yolk intake, perceived eating difficulty, vitamin D supplement, hours spent outdoors per week, poor head circumference growth.
