# Supplementary material for: Development of a nutritional risk screening tool for preterm children in outpatient settings during a complementary feeding period: a pilot study
Source: BMC Pediatr. 2022 Dec 7;22:702. doi: 10.1186/s12887-022-03774-5 (PMC9730637; doi:10.1186/s12887-022-03774-5)
Supplement: Supplementary file 6 — Additional file 6: Appendix 6–1. Univariate analysis of responses to the screening tool and the z-scores classification of body weight, length and head circumference for preterm children at the corrected age of 12–36 months [n (%)]. Appendix 6–2. Binary logistic regression analysis of responses to the screening tool and the z-scores classification of body weight, length and head circumference for preterm children at the corrected age of 12–36 months (P > 0.900 were not shown). [file 12887_2022_3774_MOESM6_ESM.docx]

Appendix6-1 Univariate analysis of responses to the screening tool and the z-scores classification of body weight, length and head circumference for preterm children at the corrected age of 12-36 months [n (%)]

|  | **WTZ≥-2** | **WTZ<-2** | **P** | **LGZ≥-2** | **LGZ<-2** | **P** | **HCZ≥-2** | **HCZ<-2** | **P** |
| --- | --- | --- | --- | --- | --- | --- | --- | --- | --- |
| n | 108 | 7 |  | 108 | 7 |  | 110 | 5 |  |
| BWTZ≥-1 | 88(81.48) | 4(57.14) | <0.001 | 88(81.48) | 4(57.14) | 0.005 | 91(82.73) | 1(20.00) | <0.001 |
| BWTZ-1~-2 | 18(16.67) | 0 |  | 17(15.74) | 1(14.28) |  | 16(14.54) | 2(40.00) |  |
| BWTZ<-2 | 2(1.85) | 3(42.86) |  | 3(2.78) | 2(28.57) |  | 3(2.73) | 2(40.00) |  |
| BLGZ≥-1 | 75(69.44) | 3(42.86) | 0.001 | 75(69.44) | 3(42.86) | 0.336 | 78(70.91) | 0 | 0.001 |
| BLGZ-1~-2 | 28(25.93) | 1(14.28) |  | 26(24.07) | 3(42.86) |  | 26(23.64) | 3(60.00) |  |
| BLGZ<-2 | 5(4.63) | 3(42.86) |  | 7(6.48) | 1(14.28) |  | 6(5.45) | 2(40.00) |  |
| BHCZ≥-1 | 85(78.70) | 3(42.86) | <0.001 | 85(78.70) | 3(42.86) | 0.095 | 88(80.00) | 0 | <0.001 |
| BHCZ-1~-2 | 19(17.59) | 1(14.28) |  | 17(15.74) | 3(42.86) |  | 18(16.36) | 2(40.00) |  |
| BHCZ<-2 | 4(3.70) | 3(42.86) |  | 6(5.56) | 1(14.28) |  | 4(3.63) | 3(60.00) |  |
| Current diseases |  |  | 0.930 |  |  | 0.930 |  |  | 0.930 |
| None | 93(86.11) | 7(100.00) |  | 93(86.11) | 7(100.00) |  | 93(86.11) | 7(100.00) |  |
| Neurological disorders | 3(2.78) | 0 |  | 3(2.78) | 0 |  | 3(2.78) | 0 |  |
| Cardiopulmonary disorders | 2(1.85) | 0 |  | 2(1.85) | 0 |  | 2(1.85) | 0 |  |
| Allergic diseases/Acute diseases | 10(9.26) | 0 |  | 10(9.26) | 0 |  | 10(9.26) | 0 |  |
| Red meat intake frequency |  |  | 0.499 |  |  | 0.499 |  |  | 0.223 |
| None | 4(3.70) | 0 |  | 4(3.70) | 0 |  | 3(2.73) | 1(20.00) |  |
| 1-3 days per week | 4(3.70) | 0 |  | 4(3.70) | 0 |  | 4(3.64) | 0 |  |
| 4-5 days per week | 46(42.59) | 5(71.43) |  | 46(42.59) | 5(71.43) |  | 49(44.54) | 2(40.00) |  |
| 6-7 days per week | 54(50.00) | 2(28.57) |  | 54(50.00) | 2(28.57) |  | 54(49.09) | 2(40.00) |  |
| White meat intake frequency |  |  | 0.507 |  |  | 0.797 |  |  | 0.845 |
| None | 1(0.93) | 0 |  | 1(0.93) | 0 |  | 1(0.91) | 0 |  |
| 1-3 days per week | 49(45.37) | 2(28.57) |  | 48(44.44) | 3(42.86) |  | 49(44.54) | 2(40.00) |  |
| 4-5 days per week | 47(43.52) | 5(71.43) |  | 48(44.44) | 4(57.14) |  | 49(44.54) | 3(60.00) |  |
| 6-7 days per week | 11(10.18) | 0 |  | 11(10.18) | 0 |  | 11(10.00) | 0 |  |
| Animal viscus intake frequency |  |  | 0.742 |  |  | 0.400 |  |  | 0.779 |
| None | 61(56.48) | 3(42.86) |  | 61(56.48) | 3(42.86) |  | 61(55.45) | 3(60.00) |  |
| 1-2 days per month | 9(8.33) | 1(14.28) |  | 10(9.26) | 0 |  | 10(9.09) | 0 |  |
| 1-3 days per week | 38(35.19) | 3(42.86) |  | 37(34.26) | 4(57.14) |  | 39(35.45) | 2(40.00) |  |
| 4-5 days per week | 0 | 0 |  | 0 | 0 |  | 0 | 0 |  |
| 6-7 days per week | 0 | 0 |  | 0 | 0 |  | 0 | 0 |  |
| Egg and yolk intake frequency |  |  | 0.150 |  |  | 0.736 |  |  | 0.217 |
| None | 8(7.41) | 0 |  | 8(7.41) | 0 |  | 8(7.27) | 0 |  |
| 1-2 days per month | 3(2.78) | 1(14.29) |  | 4(3.70) | 0 |  | 4(3.64) | 0 |  |
| 1-3 days per week | 17(15.74) | 3(42.86) |  | 18(16.67) | 2(28.57) |  | 18(16.36) | 2(40.00) |  |
| 4-5 days per week | 34(31.48) | 1(14.29) |  | 32(29.63) | 3(42.86) |  | 32(29.09) | 3(60.00) |  |
| 6-7 days per week | 46(42.59) | 2(28.57) |  | 46(42.59) | 2(28.57) |  | 48(43.64) | 0 |  |
| Cereal intake |  |  | 0.557 |  |  | 0.658 |  |  | 0.274 |
| <25g/d | 3(2.78) | 0 |  | 3(2.78) | 0 |  | 3(2.73) | 0 |  |
| 25-50g/d | 53(49.07) | 5(71.43) |  | 55(50.93) | 3(42.86) |  | 53(48.18) | 5(100.00) |  |
| 50-75g/d | 22(20.37) | 2(28.57) |  | 21(19.44) | 3(42.86) |  | 24(21.82) | 0 |  |
| 75-100g/d | 29(26.85) | 0 |  | 28(25.93) | 1(14.28) |  | 29(26.36) | 0 |  |
| >100g/d | 1(0.93) | 0 |  | 1(0.93) | 0 |  | 1(0.91) | 0 |  |
| Animal food intake |  |  | 0.159 |  |  | 0.756 |  |  | 0.275 |
| <25g/d | 8(7.41) | 0 |  | 8(7.41) | 0 |  | 8(7.27) | 0 |  |
| 25-50g/d | 70(64.81) | 7(100.00) |  | 72(66.67) | 5(71.43) |  | 72(65.45) | 5(100.00) |  |
| 50-75g/d | 30(27.78) | 0 |  | 28(25.93) | 2(28.57) |  | 30(27.27) | 0 |  |
| Milk intake |  |  | 0.504 |  |  | 0.127 |  |  | 0.426 |
| <400ml/d | 22(20.37) | 2(28.57) |  | 24(22.22) | 0 |  | 22(20.00) | 2(40.00) |  |
| 400-600ml/d | 69(63.89) | 5(71.43) |  | 67(62.04) | 7(100.00) |  | 71(64.54) | 3(60.00) |  |
| >600ml/d | 17(15.74) | 0 |  | 17(15.74) | 0 |  | 17(15.45) | 0 |  |
| Enough energy density | 108(100.00) | 7(100.00) |  | 108(100.00) | 7(100.00) |  | 108(100.00) | 7(100.00) |  |
| Perceived eating difficulty |  |  | <0.001 |  |  | <0.001 |  |  | <0.001 |
| Easy | 61(56.48) | 2(28.57) |  | 59(54.63) | 4(57.14) |  | 62(56.36) | 1(20.00) |  |
| Difficult | 47(43.52) | 4(57.14) |  | 49(45.37) | 2(28.57) |  | 48(43.64) | 3(60.00) |  |
| Very difficult | 0 | 1(14.28) |  | 0 | 1(14.28) |  | 0 | 1(20.00) |  |
| Vitamin D supplement (400-800IU/d) |  |  | 0.534 |  |  | 0.298 |  |  | 0.357 |
| None | 16(14.81) | 2(28.57) |  | 18(16.67) | 0 |  | 16(14.54) | 2(40.00) |  |
| 1-3 days per week | 13(12.04) | 0 |  | 11(10.18) | 2(28.57) |  | 12(10.91) | 1(20.00) |  |
| 4-5 days per week | 8(7.41) | 0 |  | 8(7.41) | 0 |  | 8(7.27) | 0 |  |
| 6-7 days per week | 71(65.74) | 5(71.43) |  | 71(65.74) | 5(71.43) |  | 74(67.27) | 2(40.00) |  |
| Hours spent outdoors per week |  |  | 0.341 |  |  | 0.002 |  |  | 0.174 |
| <1 hour | 8(7.41) | 1(14.28) |  | 6(5.55) | 3(42.86) |  | 9(8.18) | 0 |  |
| 1-3 hours | 25(23.15) | 0 |  | 25(23.15) | 0 |  | 25(22.73) | 0 |  |
| 3-5 hours | 19(17.59) | 3(42.86) |  | 19(17.59) | 3(42.86) |  | 19(17.27) | 3(60.00) |  |
| 5-7 hours | 52(48.15) | 3(42.86) |  | 54(50.00) | 1(14.28) |  | 53(48.18) | 2(40.00) |  |
| >7 hours | 4(3.70) | 0 |  | 4(3.70) | 0 |  | 4(3.64) | 0 |  |
| Vitamin A supplement (1333-1500IU/d) |  |  | 0.446 |  |  | 0.344 |  |  | 0.648 |
| None | 45(41.67) | 1(14.28) |  | 45(41.67) | 1(14.28) |  | 45(40.91) | 1(20.00) |  |
| 1-3 days per week | 22(20.37) | 2(28.57) |  | 21(19.44) | 3(42.86) |  | 22(20.00) | 2(40.00) |  |
| 4-5 days per week | 4((3.70) | 0 |  | 4(3.70) | 0 |  | 4(3.64) | 0 |  |
| 6-7 days per week | 37(34.26) | 4(57.14) |  | 38(35.18) | 3(42.86) |  | 39(35.45) | 2(40.00) |  |
| Iron supplement | 8(7.41) | 0 | 1.000 | 8(7.41) | 0 | 1.000 | 8(7.27) | 0 | 1.000 |
| Calcium supplement | 78(72.22) | 5(71.43) | 1.000 | 76(70.37) | 7(100.00) | 0.188 | 80(72.73) | 3(60.00) | 0.617 |
| Zinc supplement | 5(4.63) | 0 | 1.000 | 5(4.63) | 0 | 1.000 | 5(4.54) | 0 | 1.000 |
| Poor weight gain | 33(30.55) | 4(57.14) | 0.209 | 34(31.48) | 3(42.86) | 0.679 | 37(33.64) | 0 | 0.174 |
| Poor body length growth | 18(16.67) | 1(14.28) | 1.000 | 16(14.81) | 3(42.86) | 0.087 | 19(17.27) | 0 | 0.589 |
| poor head circumference growth | 41(37.96) | 5(71.43) | 0.114 | 41(37.96) | 5(71.43) | 0.114 | 41(37.27) | 5(100.00) | 0.009 |

WT/LG/HCZ: z-scores of body weight/length/head circumference three months after the interview; BWT/LG/HCZ: z-scores of birth weight/length/head circumference; all were analyzed by chi-square test or Fisher`s exact test.

Appendix6-2 Binary logistic regression analysis of responses to the screening tool and the z-scores classification of body weight, length and head circumference for preterm children at the corrected age of 12-36 months (P > 0.900 were not shown)

|  | **OR(95%CI)** | **P** |
| --- | --- | --- |
| **Model to predict underweight** |  |  |
| Z-score of birth weight ≥-1 | 0.024(0.002-0.348) | 0.006 |
| Z-score of birth weight -1~-2 | - | - |
| Z-score of birth weight <-2 (reference) | - | 0.024 |
| **Model to predict stunting** |  |  |
| Z-score of birth length ≥-1 | 0.076(0.002-2.880) | 0.165 |
| Z-score of birth length -1~-2 | - | - |
| Z-score of birth length <-2 (reference) | - | 0.165 |
| Vitamin D supplement (400-800IU/d) |  |  |
| None | - | - |
| 1-3 days per week | 4.019(0.069-235.672) | 0.503 |
| 4-5 days per week | - | - |
| 6-7 days per week (reference) | - | 0.930 |
| Poor body length growth | 0.180(0.006-5.146) | 0.316 |
| **Model to predict microcephaly** |  |  |
| - | - | - |

1. Model to predict underweight included factors of z-scores of birth weight/length/head circumference, milk/cereal/animal food intake, frequency of red meat intake, food energy density, perceived eating difficulty, poor weight gain.

2. Model to predict stunting included factors of z-scores of birth weight/length/head circumference, milk/animal food intake, perceived eating difficulty, vitamin D and calcium supplement, hours spent outdoors per week, poor body length growth.

3. Model to predict microcephaly included factors of z-scores of birth weight/length/head circumference, cereal/animal food intake, frequency of egg and yolk intake, perceived eating difficulty, vitamin D supplement, hours spent outdoors per week, poor head circumference growth.
